# Antimicrobial drimane sesquiterpenes and their effect on endophyte communities in the medical tree *Warburgia ugandensis*

**DOI:** 10.3389/fmicb.2014.00013

**Published:** 2014-02-07

**Authors:** Sigrid Drage, Birgit Mitter, Christina Tröls, Alice Muchugi, Ramni H. Jamnadass, Angela Sessitsch, Franz Hadacek

**Affiliations:** ^1^Division of Terrestrial Ecosystem Research, Department of Microbiology and Ecosystem Science, Faculty of Life Sciences, University of ViennaVienna, Austria; ^2^Bioresources Unit, Health & Environment Department, AIT Austrian Institute of Technology GmbHTulln, Austria; ^3^World Agroforestry Centre (ICRAF)Nairobi, Kenya; ^4^Department for Plant Biochemistry, Albrecht-von-Haller-Institute, Georg August UniveristätGöttingen, Germany

**Keywords:** endophytes, warburgia ugandensis, drimane sesquiterpene, bacteria diversity, fungi diversity

## Abstract

Metabolite profiles (GC–−MS), drimane sesquiterpenes, sugars and sugar alcohols, were compared with bacterial and fungal endophyte communities (T-RFLP, DNA clones, qPCR) in leaves and roots of the pepper bark tree, *Warburgia ugandensis* (*Canellaceae*). Ten individuals each were assessed from two locations east and west of the Great Rift Valley, Kenya, Africa, which differed in humidity and vegetation, closed forest versus open savannah. Despite organ- and partially site-specific variation of drimane sesquiterpenes, no clear effects on bacterial and fungal endophyte communities could be detected. The former were dominated by gram-negative *Gammaproteobacteria, Pseudomonadaceae* and *Enterobacteriaceae*, as well as gram-positive *Firmicutes*; the fungal endophyte communities were more diverse but no specific groups dominated. Despite initial expectations, the endophyte community of the pepper bark tree did not differ from other trees that much.

## Introduction

*Warburgia ugandensis* Sprague [=*W. salutaris* (Bertol.f.) Chiov], the pepper bark tree belongs to the *Canellaceae*, a small family of tropical trees, all of them aromatic and most with medicinal properties. *W. ugandesis* has a restricted distribution in evergreen forests and woodland ravines of northern KwaZulu-Natal, Swaziland, Mpumalanga, Uganda, and Kenya. This species is widely used in traditional medicine within local communities in Eastern Africa, known to cure several ailments such as stomach-ache, constipation, toothache, common cold, cough, fever, muscle pains, weak points, measles, and malaria (Beentje and Adamson, 1994; Kokwaro, 2009). Previous phytochemical studies led to the isolation of a series of unique drimane sesquiterpenes. The biological activity of the drimane sesquiterpenoids is well documented and includes antimicrobial, antifungal, insect antifeedant, cytotoxic, molluscicidal, plant growth regulation, and skin irritant effects (Jansen and De Groot, 2004). Water extracts of *W. ugandensis* elicited antibacterial activity against *Escherichia coli* and *Staphylococcus aureus* and antifungal activity against *Candida albicans* (Oilila, 2001). Preliminary phytochemical analysis revealed qualitative as well as quantitative differences in the drimane sesquiterpene profiles of individual trees grown at the same location as well as of the different organs of one tree. Consequently, the pepper bark tree represents an interesting model to explore relationships between microbial endophytes and host plant secondary metabolites, not only in terms of obtaining insights on how those interactions affect biodiversity and community composition, but also in terms of how the content of active constituents in plants that are used in traditional medicine—drimane sesquiterpenes from *Warburgia* are even considered as anti-malaria drugs (Were et al., 2010; Wube et al., 2010)—can be affected by colonization with endophytic microbes.

For this study, two populations of *W. ugandensis*, one located west (Kitale) and one east (Rumuruti) of the Great Rift Valley, Kenya, Africa, were chosen. Kitale forest is a tropical area in western Kenya situated between Mount Elgon and the Cherengani Hills at an elevation of around 2000 m above sea level. The Rumuruti forest is a dry upland forest at an elevation of 1700–2000 m above sea level. Both differ in their humidity as documented by annual rainfall amounts. Based on AFLP comparisons, the two *Warburgia* populations were recently suggested to constitute two different species as a consequence of allopatric speciation, which also occurs for species of other genera that occur both west and east of the Great Rift Valley (Muchugi et al., 2008). At both locations, leaves and roots of ten individuals were sampled. Fruits only were available at the Rumuruti site and also included into the study. We performed a polyphasic approach combining a concomitant chemical analysis of secondary metabolites in *W. ugandensis* and cultivation-independent analysis of microbes colonizing this tree.

The literature suggests that interactions between endophytes and *Warburgia* secondary metabolites should to be expected as not neutral (Carter et al., 1999; Schulz and Boyle, 2005; Saunders and Kohn, 2009). Following those assumptions we predict that:

1. Bacterial and fungal communities will resemble each other in both localities due to the selection of resistant and dominating genotypes.
2. Specific drimane sesquiterpene patterns will correlate with the presence of specific members of the endophytic microbial community, either due to their tolerance against host plant drimane sesquiterpenes or involvement in their biotransformation.
3. Drimane sesquiterpene diversity will correlate with microbial community diversity.

If, by contrast, the interactions are of more stochastic nature, we predict that

1. Bacterial and fungal communities will vary between individuals and localities with no recognizable clustering in terms of plant organ and study site.
2. No correlations will exist between the presence of specific strains in the microbial assemblage and the dominance of specific drimane sesquiterpenes in the profile.
3. Drimane sesquiterpene diversity will not correlate with microbial community diversity.

## Materials and methods

### Plant material

Leaves and roots, and, if available, fruits of pepper bark trees, *Warburgia ugandensis* Sprague, from 10 different individuals, were accessed from two distinct sites in Kenya, the first one west of the Great Rift Valley near the village Rumuruti, Marnanet North forest (1845 m. a. s. l., 0°16′ N/36°31″ E, 29.09.2007), the second one east of the Great Rift Valley in Kitale forest near the town Kitale (1900 m. a. s. l., 01°00′ N/35°01′ E, 2.10.2007), west of the Great Rift Valley. Kitale is located in a moist savannah in western Kenya situated between Mount Elgon and the Cherengani Hills at an elevation of around 2000 m. a. s. l. and has an average annual precipitation of 1269 mm; Rumuruti is located in a savannah with *W. ugandensis* mostly growing in or close to moist ravines embedded in semi-arid land at an elevation of 1700–2000 m. a. s. l. in the Laikipia district situated northwest of Mount Kenya with 739 mm average annual precipitation (http://www.climatedata.eu) (Kindt et al., 2005). Both sites are natural forests, which are managed by the Forest Department of Kenya. Voucher specimens are deposited in the University of Göttingen Herbarium (GOET), encoded K1–K10 (Kitale) and R1–R10 (Rumuruti) respectively.

Roots and leaves dedicated for DNA analysis were surface-sterilized (Sessitsch et al., 2002) and embedded in 1.5% (w/v) agar supplemented with mineral salt as used for plant tissue cultures (Murashige and Skoog, 1962). This step was performed in attempts to preserve the plant material during the transport to Austria. Roots and leaves dedicated to chemical analyses were packed into paper bags and dried in an incubator at 40°C to prevent rot during transport.

### Metabolite extraction and analysis

Three grams dried and pulverized plant tissue (leaves, fruits, and roots) were extracted with 80 ml methanol for 24 h at ambient temperature. The extract was filtered (MN 615; Macherey-Nagel, Düren, Germany) and concentrated under vacuum. Two hundred mg crude extract were fractioned over Amberlite XAD-1180 (Fluka, Buchs, Switzerland). Glass columns (15 mm diameter) were filled with 20 g resin and prepared according to the manufacturer's guidelines. Two 50 ml fractions were eluted, one with water, one with absolute ethanol. The evaporated eluates were dissolved in 10 ml methanol and stored at −20°C until further use. All used solvents were at least p.a. quality. This procedure was performed for all extracts to obtain fractions that could be analyzed in terms of drimane sesquiterpene composition. The crude extracts contained high quantities of sugar alcohols, specifically mannitol. Metabolite quantitation only was performed with the ethanol fraction, in which the drimanes were accumulated but which still contained notable amounts of sugars and sugar alcohols. A clean separation proved impossible. Consequently, the ethanol fraction represented the only fraction that provided a dataset allowing a relative comparison of drimane sesquiterpenes, fatty acids and sugar alcohols, assuming that lower sugar alcohol or sugar amounts present in the ethanol fraction represent a lower ethanol—drimane sesquiterpene ratio in the crude extract.

For GC–MS measurements, 100 μg of the dried ethanolic eluate (Amberlite XAD fractionation were dissolved in 100 μl N-methyl-N-TMS-trifluoroacetamide (MSTFA, Thermo Scientific, Waltham, MS, USA) for derivatisation into trimethylsilyl ethers. One μl of this solution was injected into an AutoSystem XL gas chromatograph (Perkin Elmer, Waltham, MS, USA) in the splitless mode, the injector temperature was 250°C. The column was a Zebron 5 ms column (18 m × 0.18 mm, 0.18 μm film thickness; Phenomenex, Torrance, CA, USA), the helium follow rate 0.8 ml/min. The temperature gradient started at 70°C and, after 3 min, rose to 300°C at a rate of 3°C/min. The gas chromatograph was linked to a TurboMass™ quadrupole mass analyzer (Perkin Elmer, Waltham, MS, USA); the transfer line temperature was set to 280°C, the ion source to 200°C, the filament current to 70 eV. The mass spectrometer was run in the TIC mode from 40–620 amu. The obtained chromatograms were integrated with TurboMass 4.1.1 (Perkin Elmer, Waltham, MS) and the peak areas were expressed as relative amounts of the total peak area (100%). The majority of drimane structures were identified on basis of a tentative fragmentation pattern analysis of the silylated derivatives. Published structures, both from natural sources and from synthesis, served as templates for spectrum interpretation.

### DNA extraction

Prior to isolation of microbial community DNA, microbial cells were dislodged from plant tissue as previously described (Reiter and Sessitsch, 2006). Therefore, leaves and roots were pulled out carefully of the agar and the remaining agar was removed thoroughly in sterile conditions. Surface disinfected fruits were cut open and the pulp was removed with a sterile spoon. DNA was isolated using the Fast DNA SPIN for Soil Kit (MP Biomedicals, Solon, OH,) as described by the manufacturer with the following modifications. Bacterial pellets were re-suspended in Na_2_PO_4_ buffer, MT buffer was added and everything was transferred to the lysing matrix E tube followed by 30 s bead-beating with a bead beater (FastPrep FP 120, Bio101, Savant Instruments, Inc., Holbrook, NY).

### T-RFLP analysis

Bacterial and fungal endophyte community profiles were examined by T-RFLP. Endophytic 16S rRNA genes were PCR-amplified using the primers 799F (5′-AAC(AC)GGATTAGATACCC(GT)-3′) (Chelius and Triplett, 2001) and 1520R (5′-AAGGAGGTGATCCAGCCGCA-3′) (Edwards et al., 1989), which was labeled with 6-carboxyfluorescein at the 5′ end. Partial fungal rRNA genes were PCR-amplified using the primers ITS1F (5′- CTTGGTCATTTAGAGGAAGTAA-3′) (Gardes and Bruns, 1993), which was labeled with 6-carboxyfluorescein at the 5′ end and ITS4 (5′- CGCCGTTACTGGGGCAATCCC -3′) (White et al., 1990). For detailed description of PCR conditions see supplementary information.

T-RFLP and data collection has been done as described by Szukics et al. (2010). The analysis of the T-RFLP profiles (identification of peaks and binning of the different fragments lengths) was done by making use of the R functions available at http://www.ibest.uidaho.edu/tools/trflp_stats/index.php(Abdo et al., 2006).

### DNA clone libraries

Ribosomal DNA libraries were constructed from a pool of aliquots of all DNA samples as well as selected plant samples making use of the Strata Clone PCR Cloning Kit (Stratagene, Agilent Technologies, Santa Clara, CA, USA) and the StrataClone SoloPack *E. coli* competent cells (Agilent Technologies, Santa Clara, CA, USA) according to the manufacturer's instructions. For a more detailed description of the cloning procedure see supplementary information. Clones have been sequenced with the primer M13f and/or M13r making use of the sequencing service of LGC Genomics (Berlin, Germany). Retrieved sequences were visualized and vector sequences were removed with sequence alignment editor package of BioEdit (Ibis Biosciences, Carlsbad, CA, USA). For identification sequences were subjected to the Basic Local Alignment Search Tool (BLAST) analysis with the National Center for Biotechnology Information (NCBI) database.

### Real-time PCR

*Pseudomonadaceae*-, *Enterobacteriaceae*- and *Firmicutes*-specific 16S rRNA genes within selected plant samples were analyzed in more detail by real-time PCR. Following primers were as used: *Pseudomonadaceae*; 8f: 5′-AGAGTTTGATCCTGGCTCAG-3′ (White et al., 1990) and PSMgX: 5′-CCTTCCTCCCAACTT-3′ (Braun-Howland et al., 1993); *Enterobacteriaceae*; En-Isu-3F: 5′-TGCCGTAACTTCGGGAGAAGGCA-3′ and En-Isu-3R: 5′-TCAAGGACCAGTGTTCAGTGTC-3′ (Matsuda et al., 2009) and *Firmicutes* 5′-CAGCAGTAGGGAATCTTC-3′ and 5′-CCGCGGTAATACGTAGGT-3′ (Pfeiffer et al., 2014). Automated analysis of PCR amplicon quantities was performed using the iCycler Optical System Software Version 3.1 (Bio-Rad Laboratories). A more detailed description is given in supplementary information.

### Statistics

Metabolite and endophytic T-RFLP patterns were analyzed by multidimensional scaling analyses (MDS) employing Bray–Curtis similarity as resemblance measure; similarity boundaries were determined by group average clustering of the Bray–Curtis similarity matrix. SIMPER analyses were performed to identify variables that contribute to similarities and dissimilarities of defined sample groups (Clarke, 1993). Diversity indices were calculated by using Fisher's alpha diversity (Fisher et al., 1943). All these analyses and their respective visualizations were performed with Primer 6 (Primer-E Ltd., Plymouth, UK). For further multivariate analyses, PCA and PLS regression, SIMCA-P 11 (Umetrics AB, Umeå, Sweden), was employed.

Parametric analyses of variance (ANOVA), provided normal distribution and variance homogeneity was given, or non-parametric Kruskal–Wallis analysis of variance, in case the above mentioned criteria failed, were carried out with respective multiple range tests (Scheffe, Bonferroni). These and simple linear regression analyses (minimum *n* = 3) were performed with Statgraphics Centurion XV (Statpoint Technologies, Inc., Warrenton, VA, USA).

### Nucleotide sequence numbers

The nucleotide sequences determined in this study have been deposited in the GenBank database; accession numbers will be provided as soon as possible.

## Results

### Metabolites, specifically drimane sesquiterpenes, characterize organs

GC–MS analyses identified 173 analytes in the extracts from 10 root, 10 leaf, and 5 fruit accessions, the latter originating only from the locality Rumuruti (R1, R3, R4, R6, R10), of which 141 were assigned as terpenoids on basis of their fragmentation patterns, *m/z* of 91, 93, 103, 105, 109, 115, 117, 119, 120, 122, 129, 131, 133, and 135, indicating the presence of a largely oxygen-free saturated ring system. These fragments do not occur in this combination in spectra of other metabolites that are usually detected by GC–MS profiling of trimethylsilylated plant metabolites, such as mono-, di-, and trisaccharides (13), sugar alcohols (4), fatty acids (6) and glycerol, which also were detected. Only one triterpene was detected, β-sitosterol.

**Figure 1:**
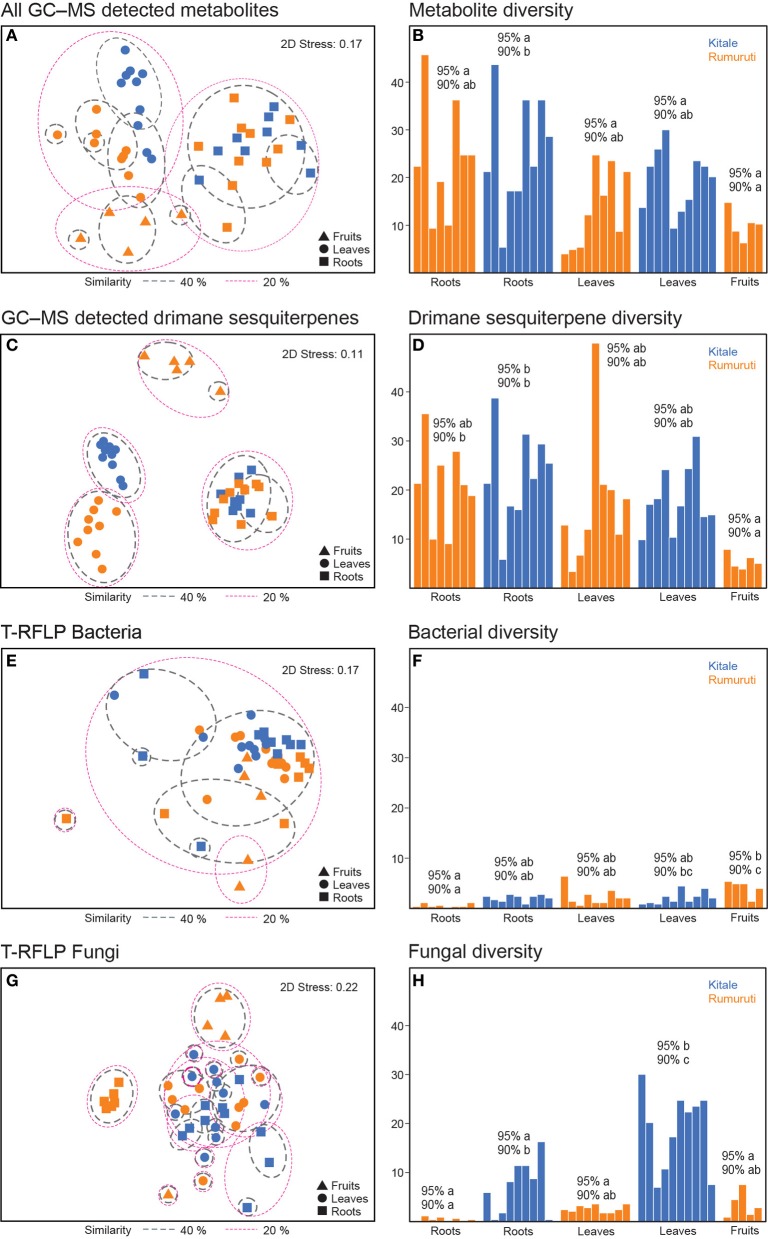
**Similarity and diversity of metabolites and endophyte communities of *Warburgia ugandensis***. All GC-MS detectable metabolites: MDS **(A)**, Fishers's α **(B)**, drimane sesquiterpes: MDS **(C)**, Fishers's α **(D)**, bacterial T-RFLP (16S rRNA): MDS **(E)**, Fishers's α **(F)**, fungal T-RFLP (ITS1, ITS4): MDS **(G)**, Fishers's α **(H)**, accessions from two localities: Kitale (blue), Rumuruti (orange); levels of significance: 95% Bonferroni; leaves and roots (*n* = 10), fruits (*n* = 5).

**Figure 2:**
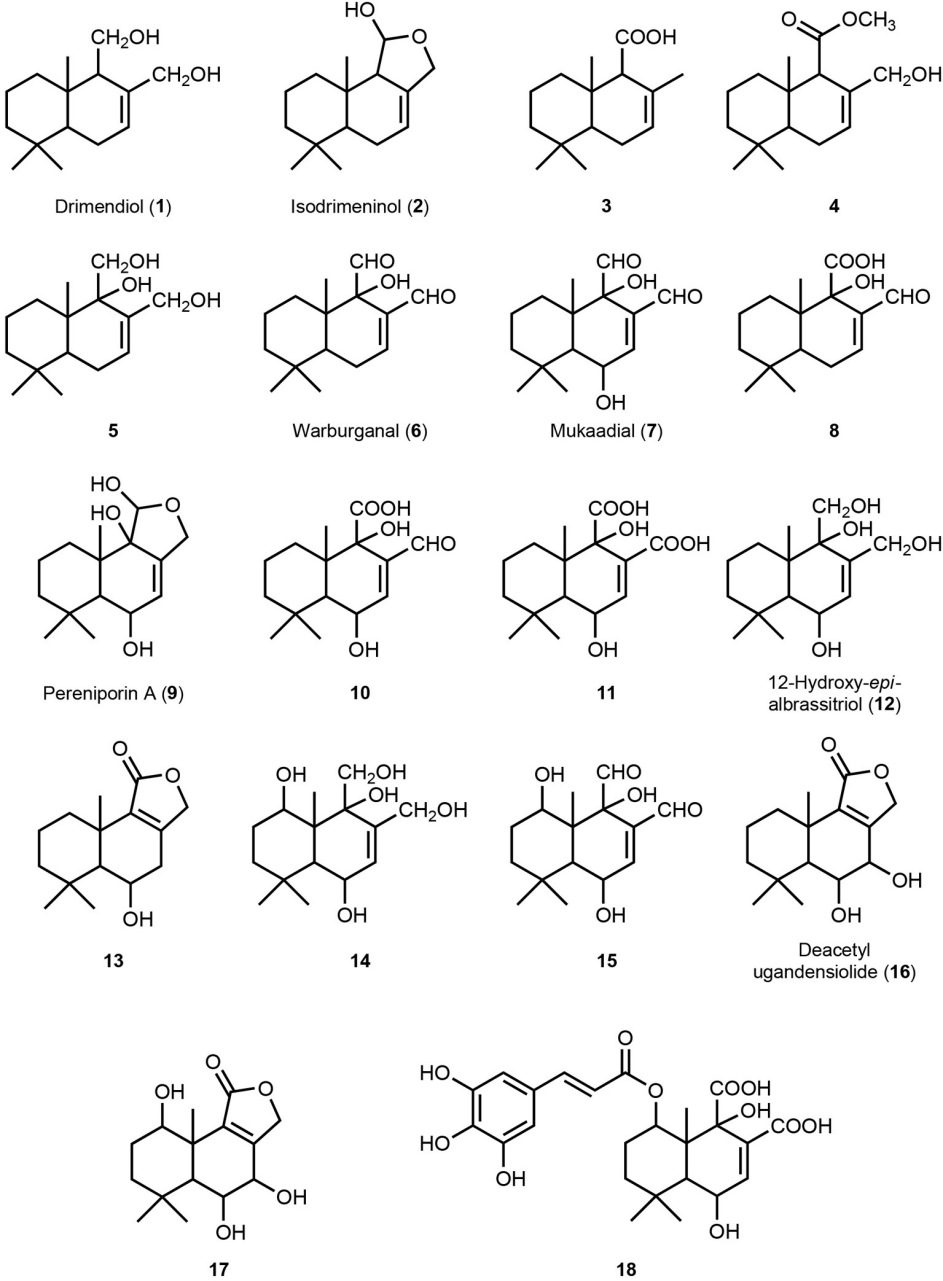
**Drimane sesquiterpene structures**. All structures are identified on basis of retention time comparison and MS fragment interpretation obtained in the GC-MS analysis (for details see Supplementary Data S1).

A multivariate analysis, MDS (non-metric multidimensional scaling) of a Bray–Curtis resemblance matrix, revealed an organ-specific clustering (Figure 1A) that was more pronounced when only drimane sesquiterpenes were included in the analysis (Figures 1C, 2D stress improving from 0.17 to 0.11). The two localities only differed in their leaf profiles; the root profiles overlapped and fruits also showed different patterns but unfortunately were available only from one locality. Metabolite diversity in each accession, Fisher's α accounting not only for the number but also the abundance of each analyte, varied considerably (Figure 1B). Again, data set limitation to drimane sesquiterpenes increased differentiation between the accession groups (Figure 1D, see levels of significance); fruits showed the lowest diversity but highest dissimilarity (Table 1). On average, only half or less of the metabolites were shared by similar organ accessions from the same locality, the five fruit accessions only shared 34% of all analytes. The considerable metabolite variation was reflected in the low average similarity within and the high dissimilarity between the organ accessions from the two localities (Table 1). Only root profiles showed some similarity.

**Table 1:** **Similarity and dissimilarity between metabolite patterns, bacterial and fungal ebdophyte communities**.

			**Kitale**		**Rumuruti**		
			**Leaves**	**Roots**	**Fruits**	**Leaves**	**Roots**
**BACTERIA (TRFs)**							
Average similarity (%)			51	41	47	51	42
Average dissimilarity (%)	**Kitale**	Leaves	–	61	64	52	65
		Roots	61	–	69	58	65
	**Rumuruti**	Fruits	64	69	–	61	69
		Leaves	52	58	61	–	54
		Roots	65	65	69	54	–
**BACTERIA (qPCR representing TRF 135)**							
Average similarity (%)			65	39	12	32	22
Average dissimilarity (%)	**Kitale**	Leaves	–	62	91	57	73
		Roots	62	–	85	68	74
	**Rumuruti**	Fruits	91	85	–	86	
		Leaves	57	68	86	–	81
		Roots	73	74	92	81	–
**FUNGI**							
Average similarity (%)			12	17	31	26	74
Average dissimilarity (%)	**Kitale**	Leaves	–	91	98	93	98
		Roots	91	–	100	91	100
	**Rumuruti**	Fruits	98	100	–	99	100
		Leaves	93	91	99	–	97
		Roots	98	100	100	97	–
**METABOLITES**							
Average similarity (%)			50	45	34	47	40
Average dissimilarity (%)	**Kitale**	Leaves	–	90	87	75	88
		Roots	90	–	91	90	59
	**Rumuruti**	Fruits	87	91	–	85	90
		Leaves	75	90	85	–	87
		Roots	88	59	90	87	–

Average similarities (within accession) and dissimilarities (between accessions) of bacterial and fungal TRFs, qPCR (Enterobacteriaceae, Pseudomonadaceae, Firmicutes, representing the bacterial TRF 135) and GC–MS metabolite profiles (roots, leaves, n = 10; fruits, n = 5) from two Warburgia ugandensis localities from Kenya, Africa.

Besides fatty acids, sugars, and sugar alcohols—by far, mannitol was the most prominent metabolite in all samples, which had to be specifically fractioned to facilitate analysis of the drimane sesquiterpenes. The latter represented the characteristic secondary metabolites that were detected by GC–MS. Peaks that were identified contributing to similarity and dissimilarity of the accessions were subjected to a tentative structure elucidation by comparative fragment analysis. The classic phytochemical literature does not provide MS spectral information for silylated sesquiterpenes, in contrast to the majority of primary metabolites (Kopka et al., 2005). Usually, only MS spectra of the underivatized metabolites are available. The structures of all thus tentatively identified drimane sesquiterpenes are illustrated in Figure 2, numbered from **1** to **18**. Their MS spectra and the tentative interpretation of the fragmentation pattern are presented in datasheet S1: **1**, drimendiol, was originally isolated from *Drymis winteri*, also a member of the *Canellaceae* (Brown, 1994); **2**, isodrimeninol, was discovered as metabolite of the moss *Porella aboris-vitae* (Asakawa et al., 1979) and the angiosperm *Polygonum hydropiper* (Asakawa et al., 1979); **3** is known as intermediate of lanosterol synthesis (Van Tamelen et al., 1982); **4** is a synthetic precursor of the drimane sesquiterpene polygodial (Jallali-Naini et al., 1981); **5** is a precursor in the chemical synthesis of warburganal (**6**) (Nakata et al., 1980); **6**, warburganal, was first isolated from the bark of *W. ugandensis*, the tree under investigation in this study (Kubo et al., 1976); **7**, mukaadial, was also isolated from the same source as **6** (Kubo et al., 1983); **8** is unknown so far; **9**, pereniporin A, was isolated from the basidiomycete *Perenniporia medullaepanis* (Kida et al., 1986); **10** is unknown; **11** is unknown; **12**, 12-Hydroxy-6-*epi*-albrassitriol, was isolated from an *Aspergillus* strain culture (Grabley et al., 1996); **13** was isolated from the fern *Protowoodsia manchuriensis* (Tanaka et al., 1980); **14** is unknown; **15** is unknown; **16**, deacetylugandensiolide, was first isolated from the heartwood of *W. ugandensis* (Brooks and Draffan, 1969); **17** is unknown; **18** is unknown.

Root accessions from both localities, Kitale and Rumuruti, showed the lowest percentage of dissimilarity, but similarity within them also was low, 40 and 45%, respectively (Table 1). The roots were characterized by the highly oxygenated drimane sesquiterpene alcohols **12** and **14**, the dialdehyde **15**, and the lactone deacetylugandensiolide (**16**); the single amounts varied considerably and this also contributed to dissimilarity (Table 2). The leaves differed not only from the roots, but also among each other. Kitale leaves were characterized by the drimane sesquiterpene dialcohol drimendiol, the acid **3**, and the hemiacetal pereniporin A (**9**). By contrast, Rumuruti leaves showed the dialdehyde mukaadial (**7**) and the acids **10** and **11**. In fruits, which were only available from some individuals at Rumuruti, again other drimane sesquiterpenes contributed to similarity: triol **5** and the corresponding dialdehyde warburganal (**6**). By its presence, the sugar alcohol mannitol contributed to similarity in all analyzed organs; its detected concentration fluctuations, however, also added to the dissimilarity. In fruits, another sugar alcohol, was prominent, *myo-*inositol; in roots, it was the monosaccharide fructose. In aerial organs, palmitic acid was more prominent than in roots. These facts (Table 2) also contribute to the clustering shown in Figures 1A,C.

Table 2**Relevant Metabolites in the GC–MS profile**.

**Table:** 

**Bacteria**						
**LEAVES**						
**Similarity:**		**Av. abund**.	**Av. simil**.	**Simil. /*SD***	**Contr. %**	**% cum**.
**Kitale**						
	Drimendiol (**1**)	28	20	2.2	39	39
	Mannitol	11	4	0.6	7	46
	Palmitic acid	5	3	1.9	7	53
	**9**	4	2	1.7	5	58
	Fructose	7	2	0.8	4	62
	**3**	3	2	1.6	3	65
**Rumuruti**						
	Palmitic acid	15	11	2.0	23	23
	Mannitol	17	8	0.9	18	41
	Mukaadial (**7**)	11	5	1.4	11	52
	Glycerol	9	5	0.8	10	62
	**10**	3	2	2.8	5	67
	**11**	3	2	1.7	4	71
	Fructose	4	1	0.7	3	74
**Dissimilarity:**			**Av. diss**.	**Diss. /*SD***	**Contr. %**	**% cum**.
	Drimendiol (**1**)		13	1.9	18	18
	Mannitol		8	1.2	11	29
	Mukaadial (**7**)		5	1.0	7	36
	Palmitic acid		5	1.4	7	43
	Glycerol		4	1.3	6	49
	Fructose		4	1.9	5	54

**Table:** 

**FRUITS**						
**Similarity:**		**Av. abund**.	**Av. simil**.	**Simil. /*SD***	**Contr. %**	**% cum**.
**Rumuruti**						
	Warburganal (**6**)	14	7	1.5	19	19
	**5**	9	6	3.6	17	36
	Mannitol	9	4	1.3	11	47
	Palmitic acid	6	4	2.2	9	56
	*myo*-Inositol	6	3	0.7	7	63
**Dissimilarity:**			**Av. diss**.	**Diss. /*SD***	**Contr. %**	**% cum**.
**Fruits and leaves Rumuruti**						
	Mannitol		7	1.2	9	9
	Warburganal (**6**)		7	1.1	9	18
	Mukaadial (**7**)		5	1.0	7	25
	Palmitic acid		5	1.4	6	31
	Glycerol		4	1.3	5	36
	**5**		4	2.3	5	41

**Table:** 

**Bacteria**						
**ROOTS**						
**Similarity:**		**Av. abund**.	**Av. simil**.	**Simil. /*SD***	**Contr. %**	**% cum**.
**Kitale**						
	**14**	15	10	1.4	22	22
	**15**	6	4	3.1	11	33
	12-Hydroxy-6-*epi*-albrassitriol (**12**)	8	4	1.0	9	42
	Mannitol	12	3	0.5	6	48
	**17**	5	2	1.5	5	53
	Deacetylugandensiolide (**16**)	4	2	0.8	5	58
	Raffinose	3	2	1.1	4	62
	**13**	4	2	1.1	4	66
**Rumuruti**		**Av. abund**.	**Av. simil**.	**Simil. /*SD***	**Contr. %**	**% cum**.
	**15**	14	6	0.9	16	16
	**14**	11	6	1.3	15	31
	Mannitol	18	6	0.8	14	45
	12-Hydroxy-6-*epi*-albrassitriol (**12**)	5	2	0.8	6	51
	Deacetylugandensiolide (**16**)	4	2	0.8	5	56
**Dissimilarity:**			**Av. diss**.	**diss. /*SD***	**Contr. %**	**% cum**.
	Mannitol		10	0.9	17	17
	**15**		6	1.0	10	27
	**14**		5	1.4	9	36
	12-Hydroxy-6-*epi*-albrassitriol (**12**)		3	1.3	6	42
	**18**		2	0.8	4	46

Contributions (Contr.) of specific metabolites (numbers 1–18 represent drimane sesquiterpenes whose structures are shown in (Figure 2) of Warburgia ugandensis accessions from two localities in Kenya, Africa, to similarity (simil.) and dissimilarity (diss.) (SD, standard deviation,(roots, leaves, n = 10; fruits, n = 5; Av. abund., average abundance).

### Bacterial endophytes show low diversity and stochastic distribution

Cultivation-independent analyses (T-RFLP, bacterial 16S rDNA) revealed a low complexity for all accessions; only three peaks were prominent (Table 3). One peak at 153 bp was present in all accessions with a generally higher intensity in roots than in leaves and an average relative abundance of 46%. One peak (300 bp) was found in twelve root samples, mainly from Kitale, as well as in two leaf samples with an average relative abundance of 7%. Finally, another peak (142 bp) was present in all but eleven samples showing an average intensity of 14%. Consequently, Bray–Curtis similarity analysis revealed no clustering of the bacteria assemblages, both in terms of locality and plant organ (Figure 1E). Fisher's alpha was low in general and varied between 0.2 and 6.4 (Figure 1F). One-Way ANOVA and Scheffe's multiple range test indicated significant differences for Rumuruti fruits, which showed the highest diversity; the lowest was found in the roots. In Kitale, the bacterial diversity in roots and leaves was more similar. SIMPER analysis identified the peak at bp 153 as the most responsible for the similarities within the bacterial communities, and its quantitative variation contributed most to the dissimilarity of the accessions (Table 3).

Table 3**Relevant bacterial T-RFs (T-RF 153 bp was resolved further by qPCR)**.

**Table:** 

**Bacteria**						
**LEAVES**						
**Similarity:**		**Av. abund**.	**Av. simil**.	**Simil. /*SD***	**Contr. %**	**% cum**.
**Kitale**						
	153	35	34	1.7	66	66
	Pseudomonadaceae				82	
	Enterobacteriaceae				18	
	145	12	8	0.9	16	82
	147	8	3	0.6	7	89
	144	7	2	0.6	4	9
**Rumuruti**						
	153	52	45	2.0	89	89
	Pseudomonadaceae				50	
	Enterobacteriaceae				44	
	145	5	1	0.3	3	92
**Dissimilarity:**			**Av. diss**.	**Diss. /*SD***	**Contr. %**	**% cum**.
	153		9	1.3	13	13
	Pseudomonadaceae				49	
	Enterobacteriaceae				41	
	115		8	0.9	12	25
	145		8	1.2	12	37
	141		5	0.9	8	45
	147		5	0.9	7	52

**Table:** 

**FRUITS**						
**Similarity:**		**Av. abund**.	**Av. simil**.	**Simil. /*SD***	**Contr. %**	**% cum**.
**Rumuruti**						
	153	31	27	2.0	58	58
	Enterobacteriaceae				52	
	Firmicutes				48	
	115	15	4	0.4	10	68
	141	7	2	0.3	4	72
**Dissimilarity:**			**Av. diss**.	**Diss. /*SD***	**Contr. %**	**% cum**.
**Fruits and leaves Rumuruti**						
	153		16	1.6	26	26
	115		8	0.9	13	39
	Pseudomonadaceae				44	
	Enterobacteriaceae				33	
	Firmicutes				10	
	141		5	0.9	8	47
	145		3	0.7	5	50
	72		3	0.8	5	53

**Table:** 

**Bacteria**						
**ROOTS**						
**Similarity:**		**Av. abund**.	**Av. simil**.	**Simil. /*SD***	**Contr. %**	**% cum**.
	153	37	28	1.3	70	70
	Pseudomonadaceae				39	
	Enterobacteriaceae				31	
	Firmicutes				30	
	300 (Paenibacillaceae)	15	8	1.0	19	89
	145	4	1	0.5	3	92
**Rumuruti**						
	153	59	40	1.3	95	95
	Pseudomonadaceae				62	
	Firmicutes				35	
**Dissimilarity:**			**Av. diss**.	**Diss. /*SD***	**Contr. %**	**% cum**.
	153		20	1.6	31	31
	Pseudomonadaceae				46	
	Enterobacteriaceae				40	
	Firmicutes				14	
	300 (Paenibacillaceae)		7	1.1	11	42
	298		5	0.4	8	50

SIMPER analyses (Bray Curtis similarity): Contributions (Contr.) of bacterial T-RFs (bp) and qPCR (Enterobacteriaceae, Pseudomonadaceae, Firmicutes, the former two γ-Proteobacteria and the latter Bacilli, which more or less represent the T-RF at 153 bp) of Warburgia ugandensis accessions from two localities in Kenya, Africa, to similarity (simil.) and dissimilarity (diss.) (SD, standard deviation, roots, leaves, n = 10; fruits, n = 5; Av. abund., average abundance).

In order to identify the dominant genera in the bacterial assemblages we constructed a 16S rRNA gene clone library from a pool of aliquots of all DNA samples. Among 53 ribotypes five chimeric sequences, two chloroplast sequences and one mitochondrial sequence were found and excluded from further analysis. Two thirds of the clearly bacterial sequences showed at least 97% similarities to known 16S rRNA genes in the NCBI database, and 30% of the clones were distantly (92–96%) related to known species (datasheet S2). The majority (90%) of the sequences belonged to the *Gammaproteobacteria*, with 52% of the clones being *Pseudomonadaceae*, predominantely the genus *Pseudomonas*, and the rest *Enterobacteriaceae*. The remaining clones belonged to the divisions of *Actinobacteria* (5%) and *Firmicutes* (5%).

**Figure 3:**
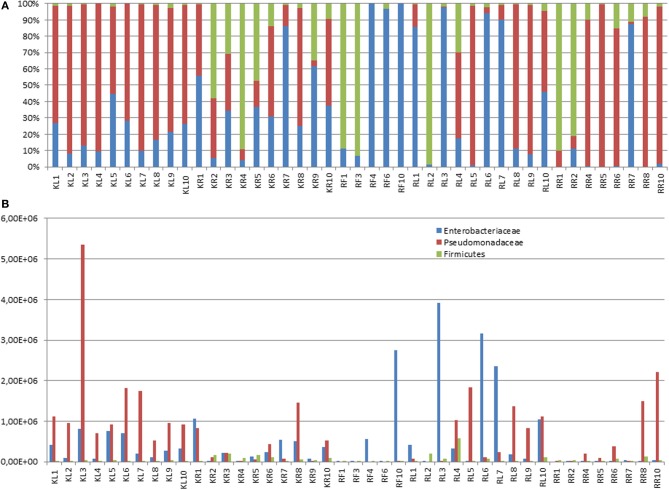
**Distribution of bacterial families in leaves and roots of *Warburgia ugandensis***. Quantiative determination of Bacili and γ-Proteobacteria (Pseudomonadaceae and Enterobacteriaceae) was carried out on basis of taxa specific quantitative PCR for all individuals (L, leaf; R, root) from each of the two accessed localities, Kitale and Rumuruti. **(A)** Relative distribution of Bacili, Pseudomonadaceae and Enterobacteriaceae describing the composition of peak at bp 153 in the 16S rDNA TRFLP analysis. **(B)** Occurrence of 16S rDNA gene copies of Bacili, Pseudomonadaceae and Enterobacteriaceae (copy numbers per ng DNA).

To gain further insights into the bacterial community in individual trees, 16S rRNA gene clone libraries from eight selected accessions, two leaf and root accessions from each locality, were constructed. About 96 clones of each cloning experiment were analyzed by RFLP profiling and sequencing. In all libraries, the majority of clones belonged to *Gammaproteobacteria* and *Bacillus* group (data not shown). Altogether five families were identified, *Pseudomonadaceae, Enterobacteriaceae, Bacillaceae, Paenibacillaceae*, and *Staphylococcaceae*, with the latter being present only in Kitale leaf accession L1. The main difference between the individual clone libraries was the varying *Pseudomonadaceae–Enterobacteriaceae* abundance ratio. Quantitative PCR detection of 16S rDNA genes specific for the taxa *Firmicutes, Enterobacteriaceae* and *Pseudomonadaceae* confirmed the clone library data. Conversely, some accessions contained mainly *Pseudomonadaceae* and hardly any *Enterobacteriaceae* or *Frimicutes* and again other accessions contained no *Pseudomonadaceae* but were dominated by *Enterobacteriaceae* or *Firmicutes*, respectively (Figure 3). In assumptions that T-RFs, which are found in a profile and in a DNA sequence, are identical if they do not differ more than in 2 bp, the T-RF at 153 bp could be assigned to the *Gammaproteobacteria* and some *Bacillaceae*. The peak at 300 bp was most probably derived from *Paenibacillaceae*. No sequence was found corresponding to the T-RF at 142 bp. The cloning and qPCR data corroborate the low complexity and relative uniformity of the bacterial T-RFLP profiles, but also indicate strong individual variation in the bacterial assemblages within individual plant tissues that is hidden within the 153 bp peak in the T-RFLP profile.

### Locality affects diversity of dissimilar fungal communities

Cultivation-independent analysis of fungal communities (T-RFLP, ITS1) in *W. ugandensis* detected 178 TRFs ranging from 35 to 500 bp in all analyzed accessions. In contrast to bacterial assemblages, fungal T-RFLP profiles varied strongly; no specific TRFs dominated the profiles, resulting in low average similarity and high dissimilarity (Table 1). Only a few common peaks were found in T-RFLPs from both sites. The Bray–Curtis similarity analysis (Figure 1G) revealed some tendencies for organ-specific clustering within roots, leaves and fruits of Rumuruti trees, but not for Kitale trees. Fungal diversity was generally higher in all Kitale accessions (Figure 1H). Root T-RFLPs showed no common peak that contributed to the similarity of accessions from the same location. Notably, Rumuruti roots were characterized by a peak at 72 bp. This TRF was found with an average abundance of 83% in the accessions and not present in those from Kitale (Table 4).

Table 4**Relevant fungal T-RFs**.

**Table:** 

**LEAVES**						
**Similarity:**		**Av. abund**.	**Av. simil**.	**Simil. /*SD***	**Contr. %**	**% cum**.
**Kitale**						
	422	8	2	0.4	17	17
	133	6	2	0.4	13	30
	139	6	2	0.4	11	41
	146	5	1	0.3	8	49
	149	3	1	0.5	5	54
	433	4	0	0.4	3	57
	41	3	0	0.3	3	60
	129	2	0	0.4	3	63
	407	3	0	0.5	3	66
	408	3	0	0.5	3	69
**Rumuruti**						
	76	13	7	1.0	29	29
	79	12	7	1.1	28	57
	469	13	4	0.4	15	72
	149	9	3	0.5	10	82
	432	2	1	0.9	4	86
**Dissimilarity:**			**Av. diss**.	**Diss. /*SD***	**Contr. %**	**% cum**.
	469		6	0.8	7	7
	76		6	1.4	7	14
	79		6	1.5	6	20
	467		5	0.4	6	26
	149		5	1.0	5	31
	422		4	0.9	5	36
	419		4	0.3	5	41
	133		3	0.7	3	44
	139		3	0.6	3	47
	129		3	0.7	3	50

**Table:** 

**FRUITS**						
**Similarity:**		**Av. abund**.	**Av. simil**.	**Simil. /*SD***	**Contr. %**	**% cum**.
**Rumuruti**						
	179	27	16	1.1	52	52
	171	25	10	0.8	33	85
	47	7	3	1.1	8	94
**Dissimilarity:**			**Av. diss**.	**Diss. /*SD***	**Contr. %**	**% cum**.
**Fruits and leaves Rumuruti**						
	179		14	1.7	14	14
	171		12	1.2	12	26
	76		6	1.4	7	33
	469		6	0.8	6	39
	79		6	1.6	6	45
	149		5	0.8	5	50

**Table:** 

**ROOTS**						
**Similarity:**		**Av. abund**.	**Av. simil**.	**Simil. /*SD***	**Contr. %**	**% cum**.
**Kitale**						
	73	8	2	0.6	15	15
	149	12	2	0.2	11	26
	75	14	2	0.2	9	35
	133	8	1	0.3	9	44
	76	6	1	0.4	8	52
	423	4	1	0.7	8	60
	140	5	1	0.3	5	65
	129	3	1	0.6	5	70
	141	5	1	0.3	5	75
	131	2	0	0.4	3	78
**Rumuruti**		**Av. abund**.	**Av. simil**.	**Simil. /*SD***	**Contr. %**	**% cum**.
	72	83	72	4.6	98	98
**Dissimilarity:**			**Av. diss**.	**Diss. /*SD***	**Contr. %**	**% cum**.
	72		41	5.0	41	41
	75		7	0.5	7	48
	149		6	0.6	6	54
	133		4	0.6	4	58

SIMPER analyses (Bray Curtis similarity): Contributions (Contr.) of fungal T-RFs (bp) of Warburgia ugandensis accessions from two localities in Kenya, Africa, to similarity (simil.) and dissimilarity (diss.) (SD, standard deviation, roots, leaves, n = 10; fruits, n = 5; Av. abund., average abundance).

In order to identify the dominant species in the fungal assemblages in *W. ugandensis* trees, we constructed an ITS region clone library from a pool of aliquots of all DNA samples. In total, 96 clones were analyzed and assigned by RFLP analysis to 47 ribotypes for which sequences were determined (datasheet S3). Three % were chimeric sequences and excluded from further analysis. The clearly non-chimeric sequences belonged to *Ascomycota* (81%) and *Basidiomycota* (16%). The two biggest classes of fungi were *Dothidiomycetes* (28%) and *Sordariomycetes* (27%), followed by *Microbotryomycetes* with 16%. The remaining sequences could be assigned to the classes of *Saccharomycetes* (12%), *Leotiomycetes* (12%), *Pezizomycetes* (2%), *Eurotiomycets* (2%), and *Tremellomycetes* (1%). The library comprised a total number of 20 fungal species; the basidiomycete *Sporidobolus ruinae* was the most abundant, representing 16% of the clones in the library. None of the identified clones unambiguously correlated with the peak at bp 72.

### Low or no correlation was found between host metabolites and endophyte communities

Explorative PLS regression (not shown) indicated only a few correlations between *Warburgia* metabolites and predominance of specific microbial community member groups, which were explored further by simple regression analyses. The identification of correlations in the data was hampered by the fact that similarity within accession groups was rather low, in many cases less than 50%. This not only applied to drimane sesquiterpenes but also to bacterial and fungal endophyte communities. Thus we defined the following criteria for acceptable correlations: (1) the correlation had to be supported by at least three cases (*n* ≥ 3); (2) reproducibility was to be at least 90% (*p* ≤ 0.1), and (3) at least 50% of all cases should support the correlation (*r*^2^ ≥ 0.5). Table 5 summarizes the results. Accordingly, the more consistently occurring metabolites, such as palmitic acid and the sugar alcohol mannitol, show higher correlation than the more variable drimane sesquiterpenes. In roots, the bacterial T-RFs and the fungal TRFs at 141 and 157 bp both correlated with palmitic acid and mannitol. Leaf endophytes, by contrast, did not correlate with palmitic acid, but with the sugars glucose and fructose and the sugar alcohol quercitol. Without exception, all correlations were positive (Table 5). On the contrary, drimane sesquiterpenes showed much fewer correlations; the majority was positive, but also three negative correlations were found, the ester **4** with the fungal TRF 141, isodrimenol **(2)** and deacetylugandensiolide (**16**) with *Firmicutes* rDNA copy numbers. The same drimanes, however, **2** and **16**, also were positively correlated, **2** with bacterial TRF 165, similarly as 12-hydroxy-*epi-*albrassitriol (**12**), and **16** with root-occurring *Enterobacteriaceae* rDNA copy numbers. The latter phenomenon was detected in both localities, Kitale and Rumuruti. Further positive correlations comprised the alcohol **5** and its oxidized derivative **8** with *Pseudomonadaceae* rRNA gene copy numbers. Moreover the metabolite diversity in *W. ugandensis* trees did not correlated.

**Table 5:**
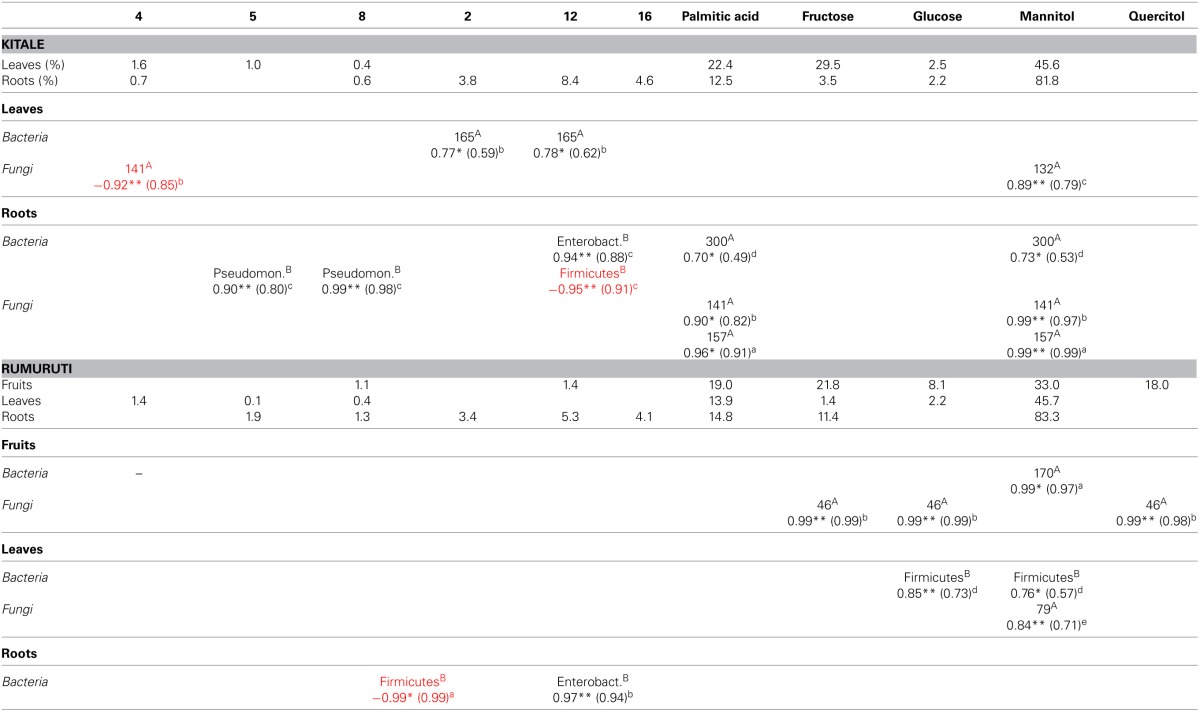
**Correlations of metabolites with microbial community components**.

Numbers represent drimane sesquiterpene structures shown in Figure 2. Occurrence (tissues and accessions, % total peak area, mean, n = 10).

^A^T-RF.

^B^qPCR, linear model y = a + bx, correlation coefficient [r^2^].

^a^n = 3.

^b^n = 4.

^c^n = 5.

^d^n = 6.

^*^p ≤ 0.10.

^**^p ≤ 0.05; Enterobact., Enterobacteriaceae; Pseudomon., Pseudomonadaceae; TRFs: bacteria: 165, not specified; 300, Paenibacillaceae; fungi: 46, not specified; 79, Penicillium sp.; 132, Cordyceps sinensis, Fimetariella rabenhorstii, Colletotrichum truncatum or Fusarium sp.; 141 and 157, not specified; negative correlations are marked in red).

With bacterial and fungal endophyte diversity, at least on basis of GC–MS and T-RFLP results (Table 6).

**Table 6:** **Correlation of metabolite with bacterial and fungal endophyte diversity**.

		***p***	***R*^**2**^**
**KITALE**			
**Leaves**			
	Bacteria	0.83	0.64
	Fungi	0.15	24.26
**Roots**			
	Bacteria	0.54	4.94
	Fungi	0.18	21.09
**RUMURUTI**			
**Leaves**			
	Bacteria	0.32	12.31
	Fungi	0.15	23.85
**Roots**			
	Bacteria	0.34	11.19
	Fungi	0.19	20.82
**ALL**			
	Bacteria	0.74	0.32
	Fungi	0.78	0.22

Fisher's a coefficients were determined for metabolite and bacterial and fungal endophyte diversity for 10 individual each from two population of Warburgia ugandensis in Kenya (Africa); ALL, correlation of all assessed data from both localities; metabolite diversity was determined by GC–MS and bacterial and fungal by T-RFLP (linear model, y = a + bx).

## Discussion

Drimane sesquiterpenes diversity turned out to be higher than that of bacterial and fungal endophyte communities, both in the leaves and roots the two accessed sites. The T-RFLP patterns of all assessed accessions did not form any specific clusters in terms of collection site and plant organ. By contrast, metabolite patterns, especially if only drimane sesquiterpenes were considered, formed organ-specific clusters. Leaf profiles differed between Kitale and Rumuruti, but root patterns were similar (Figures 1A,C). The Rumuruti leaves contained the dialdehyde mukaadial (**7**) and the corresponding acids **10** and **11**; the Kitale leaves showed drimendiol (**1**) as major compound, which represents the most reduced derivative. The co-occurring hemiacetal **7** and the simple acid **3** somehow suggest a lower degree of oxidation in Kitale leaf tissues than in those of Rumuruti. Oxidation reactions on drimane sesquiterpene structural diversity will be discussed in detail later in this text. If the more humid climate of the Kitale site, which was suggested by more intensive colonization by epiphytic lichens and ferns, caused this phenomenon can only be clarified after more study sites have been studied with a focus on this aspect.

Bacterial endophyte diversity was low in the investigated *Warburgia ugandensis* trees, and the abundant genera *Pseudomonas, Pantoea, Bacillus/Paenibacillus* represented both endophyte species frequently encountered in other plant species (Rosenblueth and Martinez-Romero, 2006). A recent review on the diversity of endophytic bacteria in forest trees (Izumi, 2011) pointed out that *Gammaproteobacteria* belong to the most prominent gram-negative bacterial endophytes, but also mentioned *Alphaproteobacteria* and *Betaproteobacteria*, which we did not detect as endophytes in *Warburgia.* The genus *Pantoea* is less often reported from tropical trees, but has been identified as endophyte in other tree genera including *Conzattia, Eucalyptus* and *Populus* (Wang et al., 2006; Izumi, 2011). The analysis of clone libraries suggested that bacterial colonization may be more a stochastic process. The high observed variability of *Pseudomonadaceae* and *Enterobacteriaceae* between the single accessions (Figure 3) that does not correlate with the composition of host secondary metabolites provides some support for this hypothesis.

In contrast to bacterial endophytes, fungi were more diverse, at least in Kitale. The T-RFLP profiles did not reveal any dominating bands in any accessed organs at any site, except perhaps for Kitale roots, which, however, were colonized by a very species-poor endophytic fungal community. The majority of the detected genera are known to occur as endophytes: *Cladosporium* (Guo et al., 2000), *Epicoccum* (Jumpponen and Jones, 2009), *Cryptococcus* (Schweigkofler and Prillinger, 1999), *Sporomiella* (Suryanarayanan et al., 1998), *Penicillium* (Narisawa et al., 2002), *Kabatiella* (Butin, 1992), *Lecythophora* (de Errasti et al., 2010), *Coniochatea* (anamorph *Lecythophora*) (Weber, 2002), *Nigrospora* (Soca-Chafre et al., 2011), *Cordyceps* (Rubini et al., 2005), *Fimetariella* (Martin-Garcia et al., 2012), *Fusarium* (Verma et al., 2011), *Neurospora* (Qi et al., 2009). Others have so far not been detected as endophytes: *Gloetinia temulenta* is a grass pathogen (Hardison, 1962); *Pseudaleuria* is a soil fungus (Xu et al., 2012); *Zopfiella latipes* is a marine fungus, but can colonize *Phragmites* (Poon and Hyde, 1998).

Contrary to initial expectations, the endophyte communities of the pepper bark tree do not differ from literature reports from other trees that much. The variation of drimane sesquiterpene profiles that was hinted in preliminary investigations was confirmed, but no substantial correlations were found with endophyte community composition. This suggests that any potential antimicrobial metabolites—drimane sesquiterpenes reportedly possess this activity (Wube et al., 2005)—do not affect the formation of an endophytic lifestyle in case of the *Warburgia* colonizers.

We may only speculate that the endophytic strains colonizing the pepper bark tree have evolved strategies to avert the toxic effects of host drimane sesquiterpenes with which they might come into contact in some stage of their life history. Due to potential hydroxyl radical formation following reduction of molecular oxygen in the presence of ferrous iron catalysts, drimane sesquiterpenes may cause oxidative stress in the affected plant tissue. A study exploring the metagenome of the rice root endophytic community identified the expression of glutathione synthase genes, a metabolite that is known to mitigate oxidative stress, as consequence of endophyte colonization amongst others (Sessitsch et al., 2012). Tolerance against oxidative stress may represent a widespread trait in soil microbes; their nutrition depends on the decomposition of organic polymers, an oxidative process that also may involve the formation of hydroxyl radicals in the Fenton reaction or the activity of oxidative enzymes (ten Have and Teunissen, 2001). The survival and growth of bacteria in a Fenton reaction milieu was demonstrated at least *in vitro* (Howsawkeng et al., 2001). Consequently, evolved tolerance to exposure to oxidative stress might help soil microbes to colonize plant tissues and help endophytes in tolerating toxic secondary metabolites of the host plant in general and in establishing populations in tissue of *W. ugandensis* trees in particular. This study, however, does not provide any information on the susceptibility of the endophytic strains against drimane sesquiterpenes. Isolation and functional characterization of endophytes of *Warburgia ugandensis* will give more insights into the strategies that have been evolved by microbes to avert the toxic effects of host drimane sesquiterpenes.

Conversely, although drimane sesquiterpenes constitute an efficient chemical defense, they may pose a threat to the producer itself by causing autotoxic effects. This is illustrated by a study showing that high amounts of acccumulated cyanogenic glycosides may cause severe autotoxic effects during strong frost periods which severe the tissue and in particular the storage compartments of the secondary metabolites that become activated by contact with sugar-cleaving enzymes (Daday, 1965). *Warburgia* tissues contain comparatively large amounts of the sugar alcohol mannitol; sugar alcohols have been shown to protect against hydroxyl radicals (Smirnoff and Cumbes, 1989). It is quite feasible assuming that the high amounts of mannitol in the pepper bark tissue are linked to the drimane sesquiterpenes and aim to keep the oxidative effects of drimanes from destroyed compartments at a tolerable level. This potential incurring of protective costs might explain why drimane sesquiterpenes are only utilized by few organisms despite their wide distribution in secondary-metabolite-producing organisms (Jansen and De Groot, 2004). Another aspect merits consideration: the microbial communities that colonize *Warburgia* tissues do not differ substantially from those reported to colonize other trees. In diverse plant communities, plants with extreme chemical defenses most probably have low effects on the assemblage of possible microbial colonization candidates. Those, which stochastically colonize *Warburgia* tissues, may be doomed, but others, which colonize also other plants, will survive, propagate and build assemblages of microbes that are able to colonize *Warburgia*. Deterministic and stochastic factors both can shape the specific composition of these assemblages in a complex and difficult-to-elucidate fashion. This also complicates and unambiguous the decision if endophytes affect the biosynthesis of and the yield of secondary metabolites in their host plants.

*Warburgia ugandensis* is a tree species with high ethnopharmaceutical relevance. In traditional local medicine the powdered bark is usually taken orally as aqueous infusion, smoked, or mixed with fat and applied externally as an ointment for treatment of a broad range of human diseases including measles and malaria (Beentje and Adamson, 1994; Kokwaro, 2009). The existence of this tree species in its natural environment is however under severe threat. Deforestation and unsustainable use (harvest of roots and barks) results in drastic loss of these trees. Knowledge of the factors determining the variation in the patterns of drimane sesquiterpenes could help to identify individuals with high yield production traits for drimane sesquiterpenes in order to identify suitable genotypes or cultivation practices for plantations of this tree. This would substantially increase the value of this tree species for local farmers and facilitate preservation programs. The genetic background of drimane sesquiterpene biosynthesis in *Warburgia ugandensis* is still poorly understood. Muge and colleagues isolated and characterized a partial gene encoding for a sesquiterpene synthase (Muge, 2008). Variations in the drimane sesquiterpene content in different plant organs of the tree may be explained partly by plant tissue specific expression of genes for secondary metabolites (Kombrink and Somssich, 1995). However the actual reasons for the strong individual variations in the drimane sesquiterpene pattern in the pepper bark tree still remain obscure.

In conclusion, our study revealed that (1) the endophyte community of the tropical tree *Warburgia ugandensis* resembles at the genus level that of trees in temperate climates; (2) the endophyte community is not shaped by host drimane sesquiterpenes; (3) the diversity of the endophytic microflora in *Warburgia ugendensis* does not correlate with that of host drimane sesquiterpenes**;** and (4) other factors rather than endophytic microbes might be responsible for the high variations in the content and composition of drimane sesquiterpenes in the pepper bark tree.

Further studies including also other tropical trees are required to explore if the conclusions from this paper can be confirmed on a broader scale. Ideally, such studies should consider the effect of climatic stress such as drought or high light exposure for the individual trees, if possible, to facilitate a more insightful assessment of the implications of variation in the analyzed microbial assemblages in geographically different host populations. Endophytes can affect the metabolism and health of their hosts in some cases, but they may fail to do so in other cases (Porras-Alfaro and Bayman, 2011). Amensalistic, commensalistic and mutualistic relations can occur. The unbiased assessment of endophytes (that report positive, negative, and null relationships) will be required to obtain insights into the potential effects of microbial endophytes on the development and metabolism of the host plant as well as contributions to its resistance against various forms of abiotic and biotic stress.

### Conflict of interest statement

The authors declare that the research was conducted in the absence of any commercial or financial relationships that could be construed as a potential conflict of interest.

## Supplementary material

Supplementary Data S1 **Tentative Structures of all Drimane Sesquiterpene Analytes from *Warburgia ugandensis***. This file provides information on which the tentative structure identification of drimane sesquiterpenes analytes is based in this study. Each analyte is presented on three pages: Page 1 contains the tentative structure with the analysis retention time, page 2 presents the MS spectrum together with the structure of the derivatized analyte, and page 3 illustrates structure fragments corresponding to specific fragments in the EI–MS spectrum. The tentative structure assignment is based on these data for each analyte respectively. The numbering corresponds to that presented in Figure 2.

Table S2 **Bacterial endophytes from leaves, fruits and roots of Warburgia ugandensis**.

Table S3 **Endophytic fungi from leaves, fruits and roots of Warburgia ugandensis**. **Material and Methods. Supplementary Information**.
