# Supplementary material for: Antimicrobial drimane sesquiterpenes and their effect on endophyte communities in the medical tree Warburgia ugandensis
Source: Front Microbiol. 2014 Feb 7;5:13. doi: 10.3389/fmicb.2014.00013 (PMC3916764; doi:10.3389/fmicb.2014.00013)
Supplement: Supplementary Data S1 — Tentative Structures of all Drimane Sesquiterpene Analytes from Warburgia ugandensis. This file provides information on which the tentative structure identification of drimane sesquiterpenes analytes is based in this study. Each analyte is presented on three pages: Page 1 contains the tentative structure with the analysis retention time, page 2 presents the MS spectrum together with the structure of the derivatized analyte, and page 3 illustrates structure fragments corresponding to specific fragments in the EI–MS spectrum. The tentative structure assignment is based on these data for each analyte respectively. The numbering corresponds to that presented in Figure 2. [file Presentation1.ZIP › Material and Methods (Supplementary Information).pdf]

Material and Methods

2.4. T-RFLP analysis

Bacterial and fungal endophyte community profiles were examined by T-RFLP. Endophytic 16S rRNA genes were PCR-amplified using the primers 799F (5´-AAC(AC)GGATTAGATACCC(GT)-3´) (Chelius and Triplett, 2006) and 1520R (5´-AAGGAGGTGATCCAGCCGCA-3´) (Edwards et al., 1989), which was labeled with 6-carboxyfluorescein at the 5´ end. Partial fungal rRNA genes were PCR-amplified using the primers ITS1F (5´- CTTGGTCATTTAGAGGAAGTAA-3´) (Gardes and Bruns, 1993), which was labeled with 6-carboxyfluorescein at the 5´ end and ITS4 (5´-CGCCGTTACTGGGGCAATCCC -3´) (White et al., 1990).

PCR-reactions (50 µl total volume) contained 10–30 ng of DNA, 1x PCR reaction buffer (Invitrogen), 1.5 mM $MgCl_2$ (16S rDNA) or 3 mM $MgCl_2$ (ITS), 0.2 µM of each primer, 0.2 mM of each deoxynucleoside triphosphate, and 2.5 U Taq DNA polymerase (LifeTech, Vienna, Austria).

PCR amplifications were performed with an initial denaturation step for 5 minutes at 95°C, 30 cycles consisting of denaturation for 30 sec at 95°C, primer annealing for 30 sec at 53°C (16S rDNA) or 1 min at 50°C (ITS), polymerization for 1 min at 72°C (16S rDNA) or 2 minutes at 72°C (ITS), and completed by a final extension for 10 min at 72°C. PCR products (5 µl) were checked by electrophoresis in 0.8% (w/v) agarose gels (Biozym Biotech Trading, Vienna, Austria). Four PCR products of each sample were pooled and 16S rDNA PCR amplicons were subjected to electrophoresis in 2% (w/v) agarose gels. The band of interest containing the PCR-product of bacterial 16S rDNA (ca. 720 bp) was excised and purified using the QIAquick™ gel extraction kit (Qiagen GmbH, Hilden, Germany). Pooled ITS PCR amplification products were purified using the QIAquick™ PCR Purification kit (Qiagen GmbH, Hilden, Germany). One hundred and fifty ng purified PCR products were digested with 5 U of the endonucelase *Hae*III (Promega GmbH, Mannheim, Germany) at 37°C for 3 h in a total reaction volume of 10 µl.

2.5. DNA clone libraries

Ribosomal DNA libraries were constructed from a pool of aliquots of all DNA samples as well as selected plant samples. PCR primers 8f: 5´-AGAGTTTGATCCTGGCTCAG-3´ (White et al., 1990) and 1520r as well as ITS1 and ITS4 were used to amplify bacterial and fungal ribosomal DNA, respectively using the same reaction mixtures and conditions as outlined for the T-RFLP analysis, except that no primer was labeled. PCR products were purified by using a QIAquick™ PCR Purification kit (QIAGEN GmbH, Hilden, Germany). DNA fragments were ligated into the vector pSC-A-amp/kan (Strata Clone PCR Cloning Kit, Stratagene, Agilent Technologies, Santa Clara, CA, USA) and the ligation products were transformed into competent *E. coli* cells (StrataClone SoloPack Competent Cells, Agilent Technologies, Santa Clara, CA, USA) according to the manufacturer’s instructions. One hundred clones per library, appearing as white colonies on indicator plates containing X-Gal (5-bromo-4-chloro-3-indolyl-β-D-galactopyranoside) and IPTG (isopropyl-β-thiogalactopyranoside) were picked and used for colony-PCR amplification with the primers M13f (5´-TGTAAAACGACGGCCAGT-3´) and M13r (5´-GGAAACAGCTATGACCATG-3´) to amplify cloned inserts. PCR was performed as described for 16S rDNA amplification, but setting the annealing temperature at 50°C. Approximately 150 ng DNA of each PCR were digested with 5 U of the endonucleases *Hae*III (Invitrogen) and *Alu*I (Promega GmbH, Mannheim, Germany), respectively. RFLP patterns were analysed by size separation of the restriction fragments by electrophoresis in 2% (w/v) agarose gels (Biozym Biotech Trading,

Vienna, Austria). One clone of each ribotype was sequenced with the primer M13f and/or M13r making use of the sequencing service of LGC Genomics (Berlin, Germany). Retrieved sequences were visualized and vector sequences were removed with sequence alignment editor package of BioEdit (Ibis Biosciences, Carlsbad, CA, USA). For identification sequences were subjected to the Basic Local Alignment Search Tool (BLAST) analysis with the National Center for Biotechnology Information (NCBI) database.

2.6. Real-time PCR

Pseudomonadaceae- and *Enterobacteriaceae*-specific 16S rRNA genes within selected plant samples were analysed in more detail by real-time PCR using an iCycler IQ (Biorad). The 25 µl PCR reaction mix contained 0.2-58ng template (depending on DNA sample, dilution and primer set), 12.5 µl of Q Mix (Biorad, 100mM KCl, 40 mM Tris-HCl, 6mM MgCl2, 0.4 mM each of dNTP, 50 U ml–1 iTaq DNA Polymerase, SYBR Green I, 20 nM fluorescein, and stabilizer) and 0.15 µM of each primer (*Pseudomonadaceae*; 8f: 5´-AGAGTTTGATCCTGGCTCAG-3´ (White et al., 1990), PSMgX: 5´-CCTTCCTCCCAACTT-3´ (Braun-Howland et al., 1993) or 0.4µM of each primer (*Enterobacteriaceae*; En-Isu-3F: 5´- TGCCGTAACTTCGGGAGAAGGCA-3´ and En-Isu-3R: 5´-TCAAGGACCAGTGTTCAGTGTC-3´ (Matsuda et al., 2009), respectively. Fluorescent acquisition was performed at 72°C for pseudomonads 16S rDNA and at 84.5°C for enterobacterial 16S rDNA, where all primer dimers had melted but specific products had not. PCR conditions for pseudomonads were 5 min at 95°C, followed by 35 cycles of 95°C for 30 sec, 1 min at 52°C and 72°C for 1 min. PCR conditions for enterobacteria were 3 min at 95°C, followed by 40 cycles of 94°C for 30 sec, 30 sec at 52°C and 72°C for 45 sec. Followed by a final elongation step of 72°C for 7 min, respectively. Melting curve analysis of the PCR products was conducted following each assay to confirm that the fluorescence signal originated from specific PCR products and not from primer-dimers or other artifacts. DNA samples were tested in duplicate 2-fold serial dilutions. Standard curves were generated using duplicate 10-fold serial dilutions of isolated plasmid DNA. Automated analysis of PCR amplicon quantities was performed using the iCycler Optical System Software Version 3.1 (Bio-Rad Laboratories). Abundances of pseudomonads and enterobacterial 16S rDNA genes refer to copy numbers per ng DNA. Reaction efficiencies of qPCRs were 83.3% (+ 0.36) for pseudomonads 16S rDNA and 90.4% (+ 4.7) for enterobacterial 16S rDNA and R2 values were 0.99 for all runs. In order to exclude inhibitory effects on qPCR amplification a serial dilution of DNA served as template for PCR reaction. Firmicute specific real time PCR has been done using the primers and conditions described by Pfeiffer and colleagues (accepted for publication). For standard preparation, amplicons of the investigated phylogenetic groups were generated using a mixture of DNA from all target plant samples as template. Therefore, PCR cocktails of 25 µl contained 30 ng DNA, 1x PCR reaction buffer, 2 mM $MgCl_2$, 0.2 mM of each dNTP, 1 mg/ml BSA, 2.5 U Taq DNA polymerase (Invitrogen) and 0.15 µM of each oligonucleotide (primer sets were the same as described above). PCR amplifications were run under the following conditions: 5 min at 95°C, followed by 30 cycles of 95°C for 30 sec, 1 min at 52°C, 72°C for 2 min, and a final elongation step for 7 min at 72°C. Products from replicate amplifications (5 µl) were checked on a 1% (w/v) agarose gel, pooled and purified using the QIAquick™ Gel Extraction Kit (Qiagen GmbH, Hilden, Germany). Purified amplicons were ligated into the StrataClone™ PCR cloning vector pSC-A (Agilent Technologies, Santa Clara, CA, USA), and StrataClone™ SoloPack® competent cells (Agilent Technologies, Santa Clara, CA, USA) were then transformed with the ligation products. Plasmid DNA was isolated using the QIAprep Spin Miniprep Kit (Qiagen GmbH, Hilden, Germany) and quantified with a NanoDrop ND-1000 Spectrophotometer (Nanodrop Technologies, Montchanin, DE, USA).
