# Supplementary material for: Antimicrobial drimane sesquiterpenes and their effect on endophyte communities in the medical tree Warburgia ugandensis
Source: Front Microbiol. 2014 Feb 7;5:13. doi: 10.3389/fmicb.2014.00013 (PMC3916764; doi:10.3389/fmicb.2014.00013)
Supplement: Supplementary Data S1 — Tentative Structures of all Drimane Sesquiterpene Analytes from Warburgia ugandensis. This file provides information on which the tentative structure identification of drimane sesquiterpenes analytes is based in this study. Each analyte is presented on three pages: Page 1 contains the tentative structure with the analysis retention time, page 2 presents the MS spectrum together with the structure of the derivatized analyte, and page 3 illustrates structure fragments corresponding to specific fragments in the EI–MS spectrum. The tentative structure assignment is based on these data for each analyte respectively. The numbering corresponds to that presented in Figure 2. [file Presentation1.ZIP › Supplementary Data S1.pdf]

S1. Supplementary Data: Tentative Structures of all Drimane Sesquiterpene Analytes from *Warburgia ugandensis*

This file provides information on which the tentative structure identification of drimane sesquiterpenes analytes is based in this study. Each analyte is presented on three pages: Page 1 contains the tentative structure with the analysis retention time, page 2 presents the MS spectrum together with the structure of the derivatized analyte, and page 3 illustrates structure fragments corresponding to specific fragments in the EI–MS spectrum. The tentative structure assignment is based on these data for each analyte respectively. The numbering corresponds to that presented in Figure 2.

Drimendiol (**1**)

t_{Ret} = 38.3 min

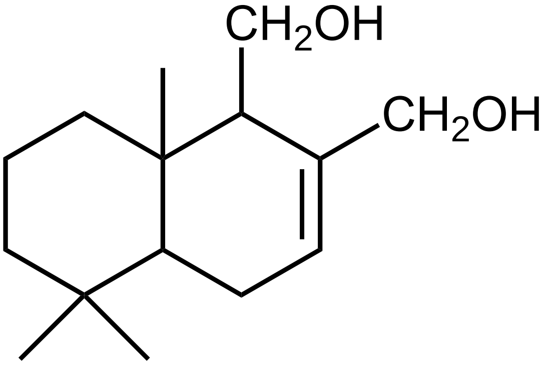

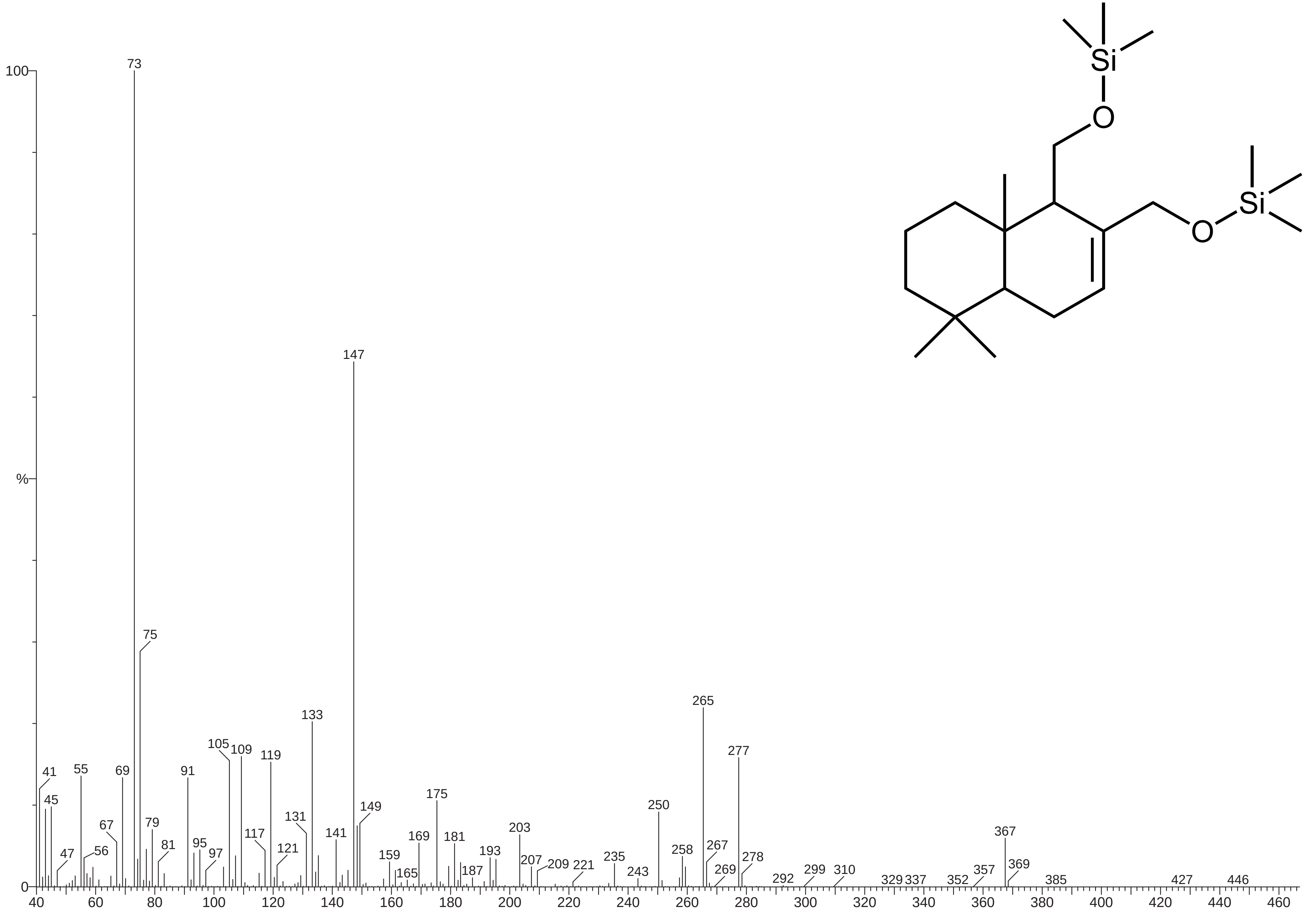

100
%
0
73
147
75
265
277
133
105
109
119
41
55
69
91
45
149
175
250
367

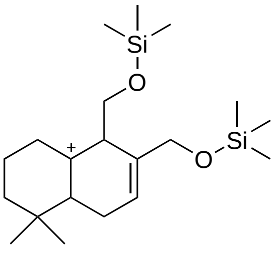

m/z = 367

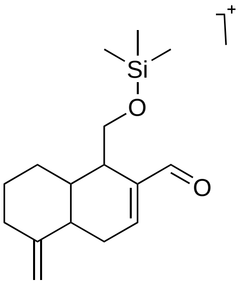

m/z = 277

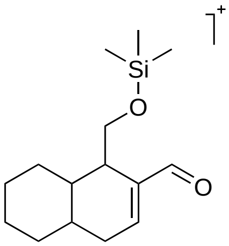

m/z = 265

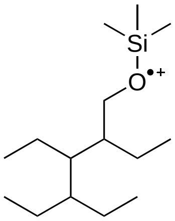

m/z = 258

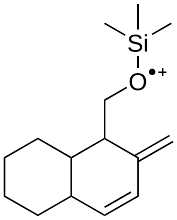

m/z = 250

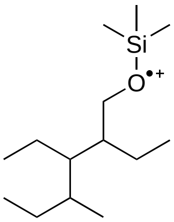

m/z = 243

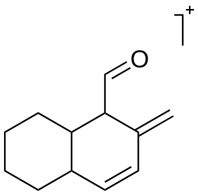

m/z = 175

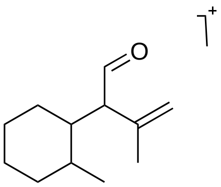

m/z = 179

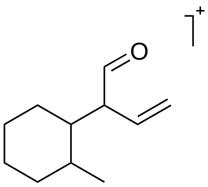

m/z = 165

Isodrimeninol (**2**)

t_{Ret} = 42.7 min

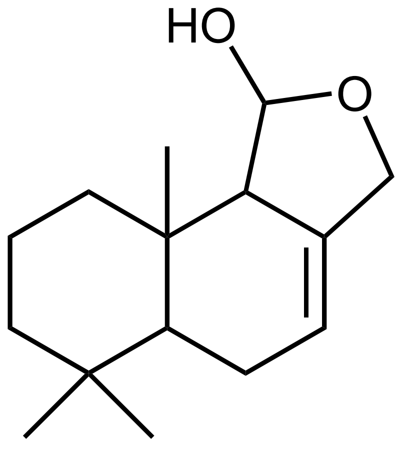

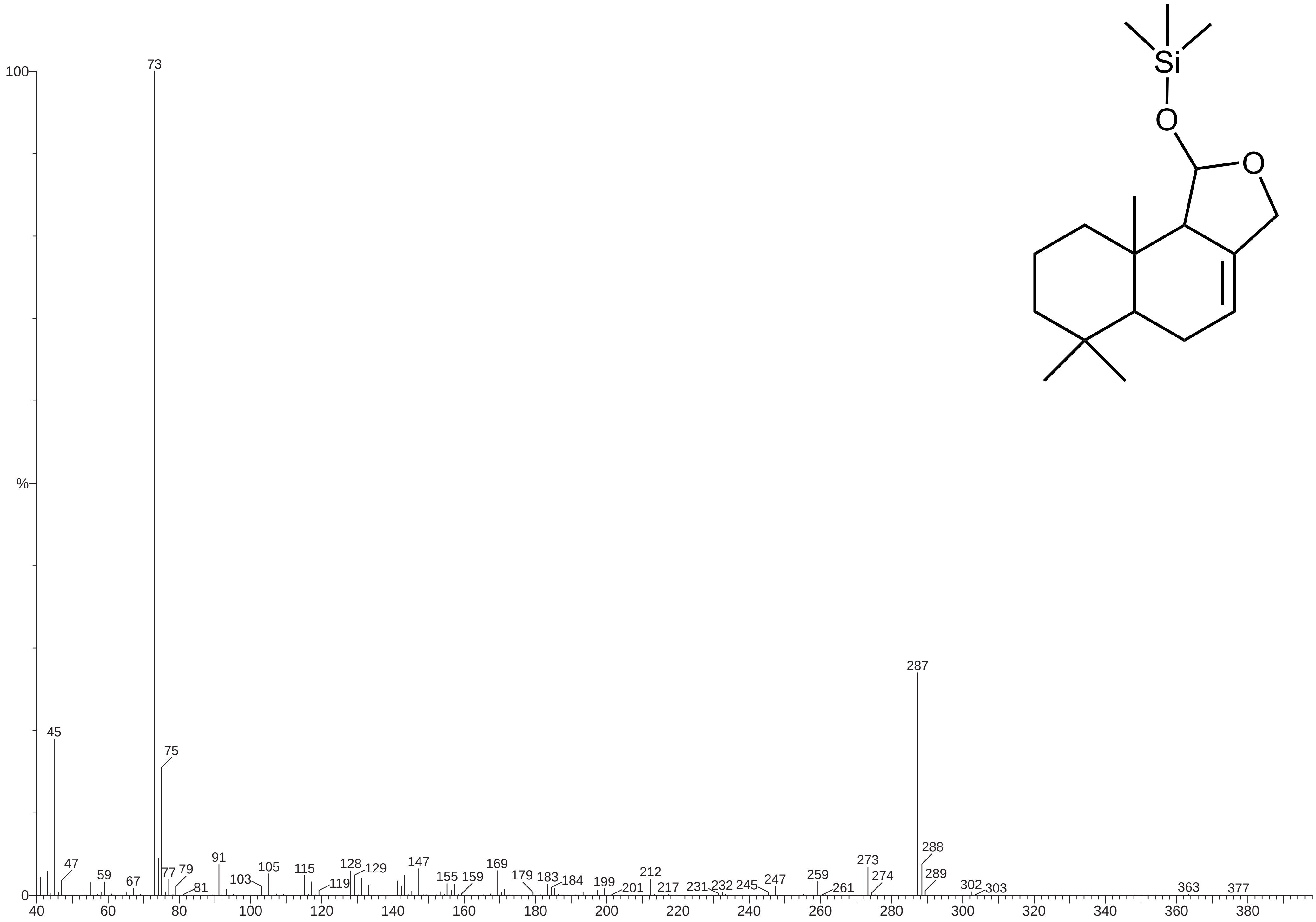

100
%
0
73
45
75
47
59
67
77
79
81
91
103
105
115
119
128
129
147
155
159
169
179
183
184
199
201
212
217
231
232
245
247
259
261
273
274
287
288
289
302
303
363
377
40
60
80
100
120
140
160
180
200
220
240
260
280
300
320
340
360
380
Si
O
O

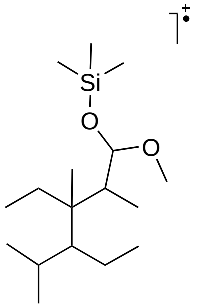

m/z = 303

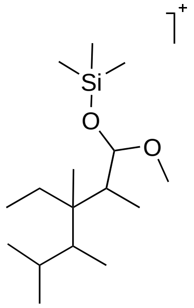

m/z = 287

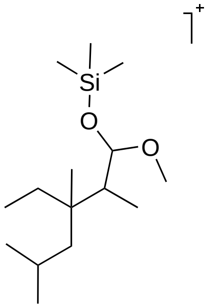

m/z = 273

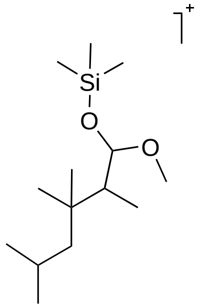

m/z = 259

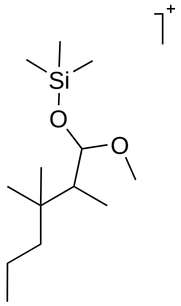

m/z = 245

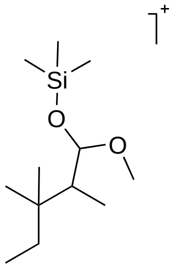

m/z = 231

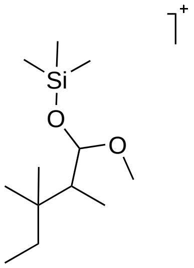

m/z = 231

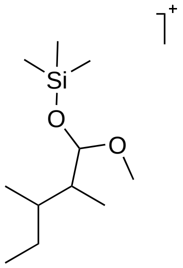

m/z = 217

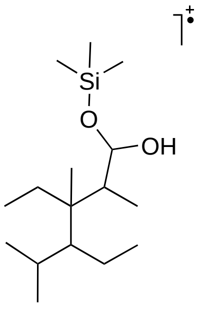

m/z = 288

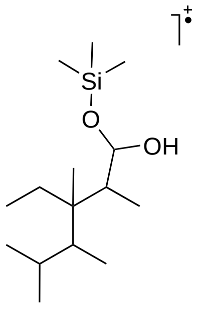

m/z = 274

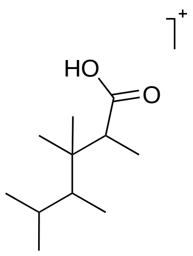

m/z = 185

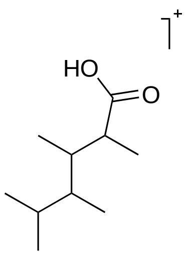

m/z = 171

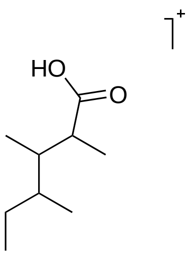

m/z = 157

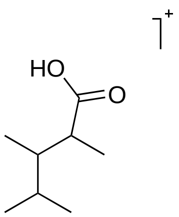

m/z = 143

3

t_{Ret} = 42.2 min

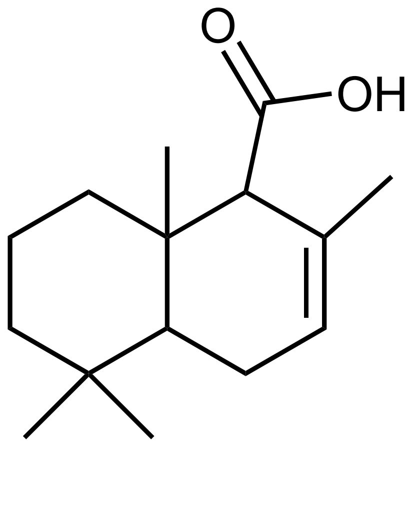

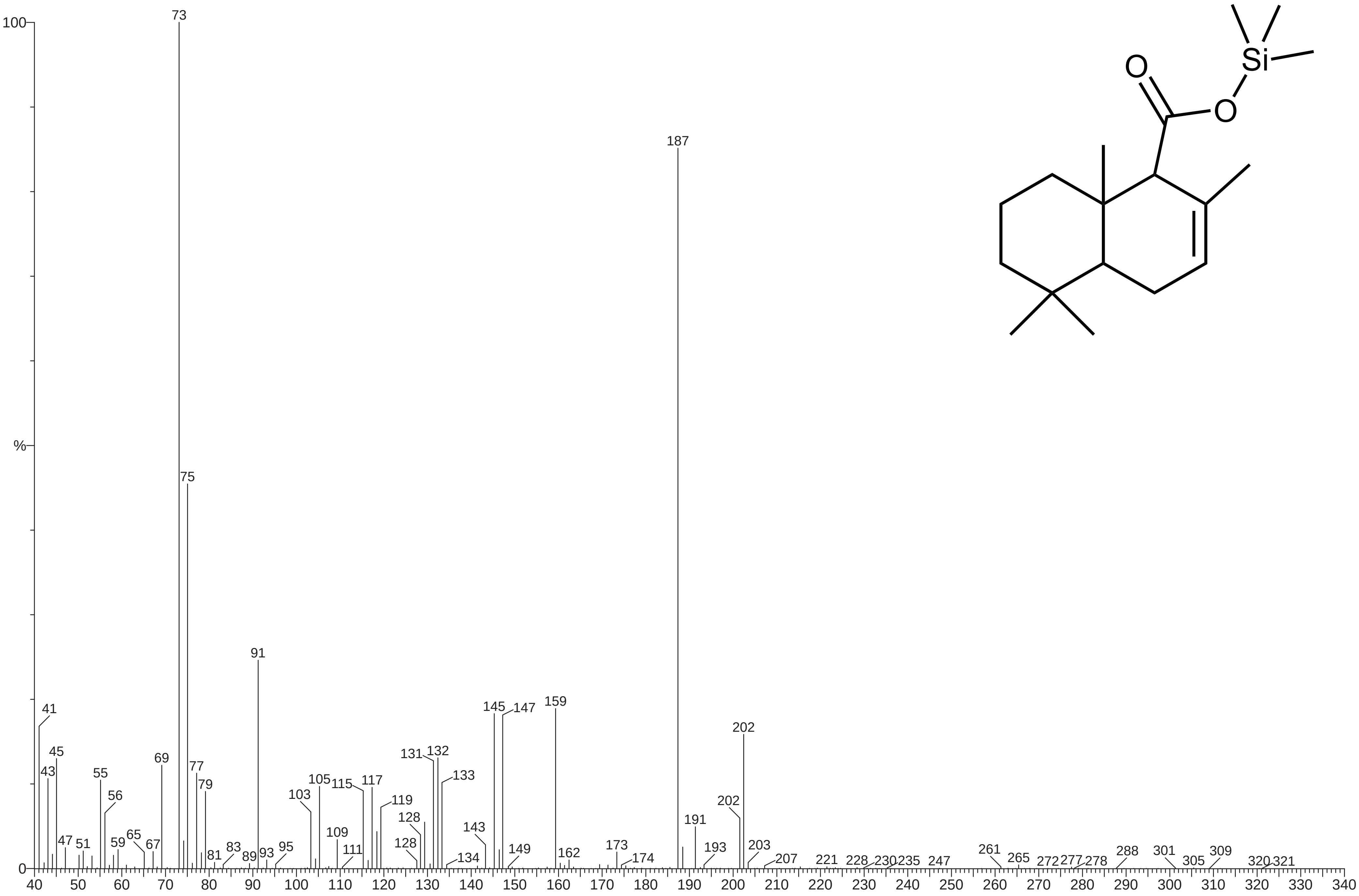
100
%
0
73
187
75
91
41
45
43
55
56
69
77
79
47
51
59
65
67
81
83
89
93
95
103
105
115
117
109
111
119
131
132
133
128
128
134
143
145
147
149
159
162
173
174
191
193
202
202
203
207
221
228
230
235
247
261
265
272
277
278
288
301
305
309
320
321
40 50 60 70 80 90 100 110 120 130 140 150 160 170 180 190 200 210 220 230 240 250 260 270 280 290 300 310 320 330 340
O
O
Si

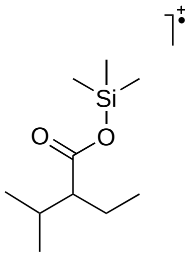

m/z = 202

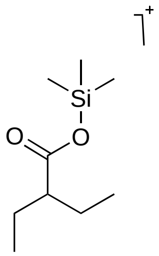

m/z = 187

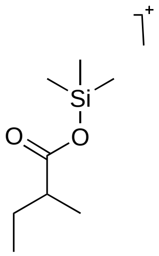

m/z = 173

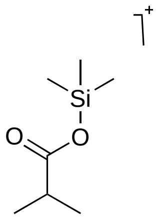

m/z = 159

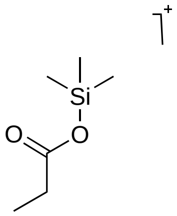

m/z = 145

4

t_{Ret} = 36.0 min

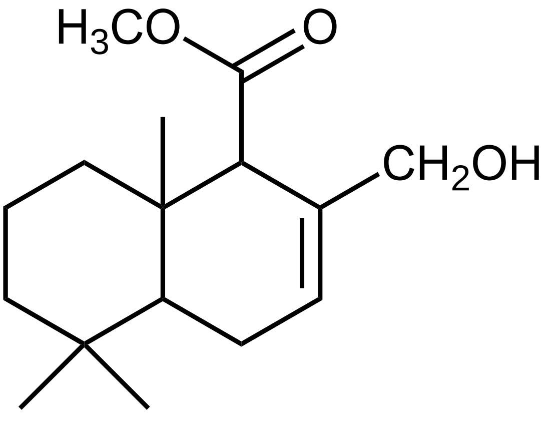

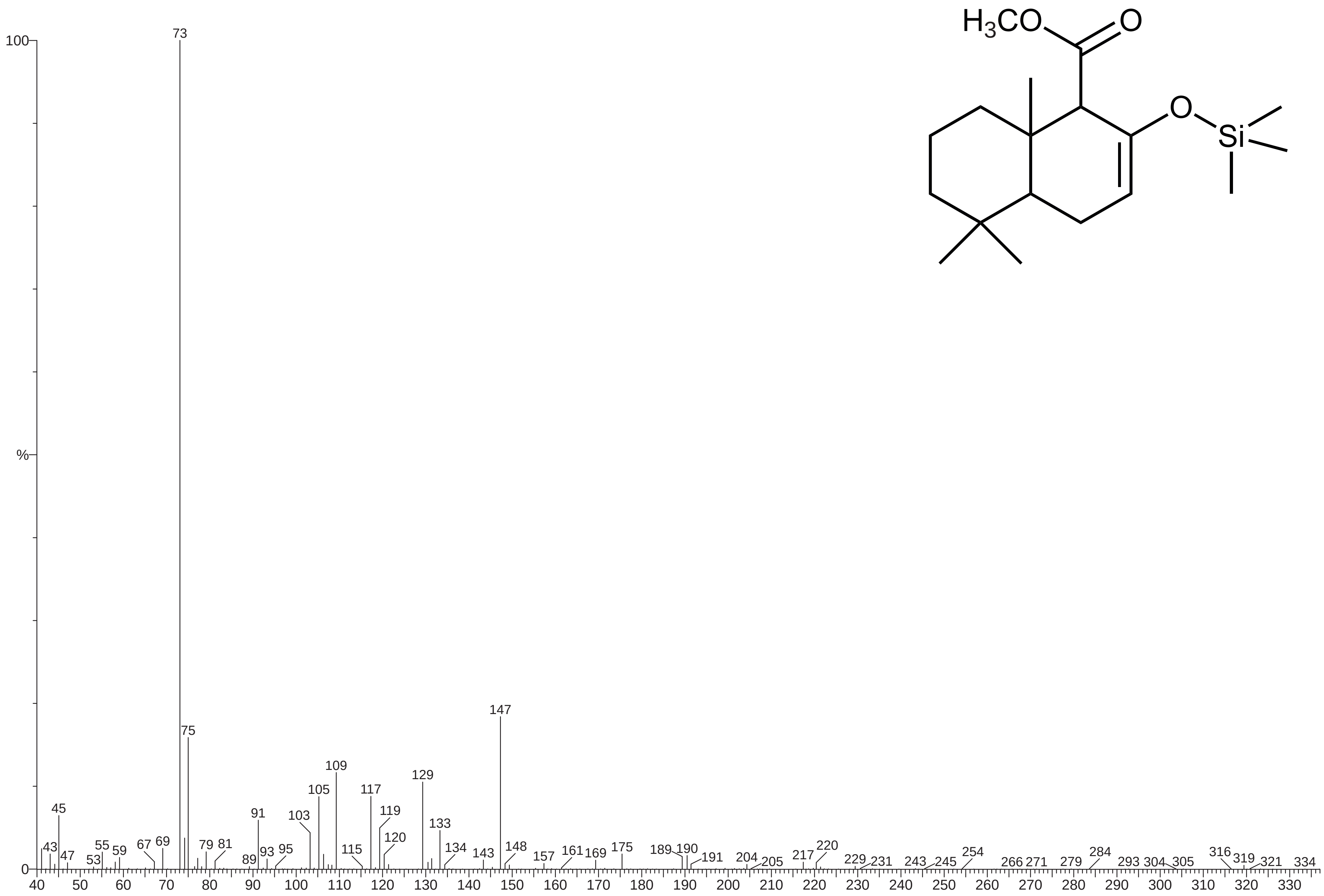

H3CO
O
O
Si
100
%
0
73
75
147
109
129
105
117
45
91
103
119
133
120
148
134
143
175
161
169
157
189
190
191
220
217
204
205
229
231
243
245
254
266
271
279
284
293
304
305
316
319
321
334
115
43
47
53
55
59
67
69
79
81
89
93
95
40 50 60 70 80 90 100 110 120 130 140 150 160 170 180 190 200 210 220 230 240 250 260 270 280 290 300 310 320 330

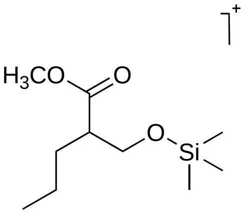

m/z = 217

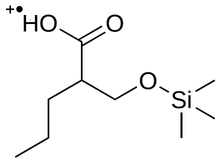

m/z = 204

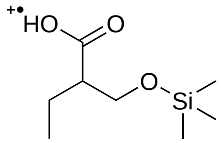

m/z = 190

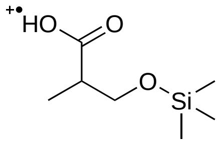

m/z = 175

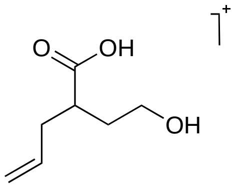

m/z = 143

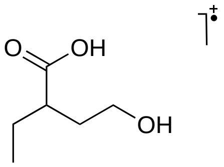

m/z = 132

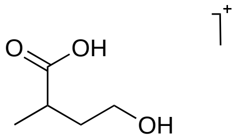

m/z = 117

5

t_{Ret} = 40.7 min

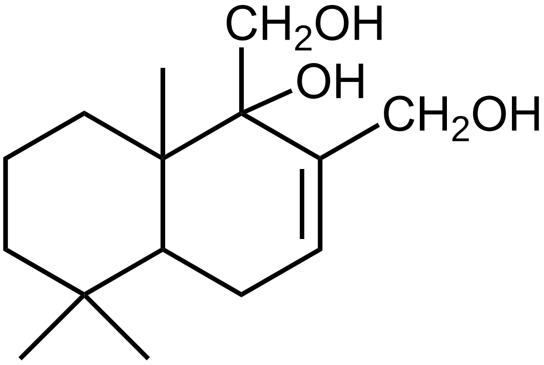

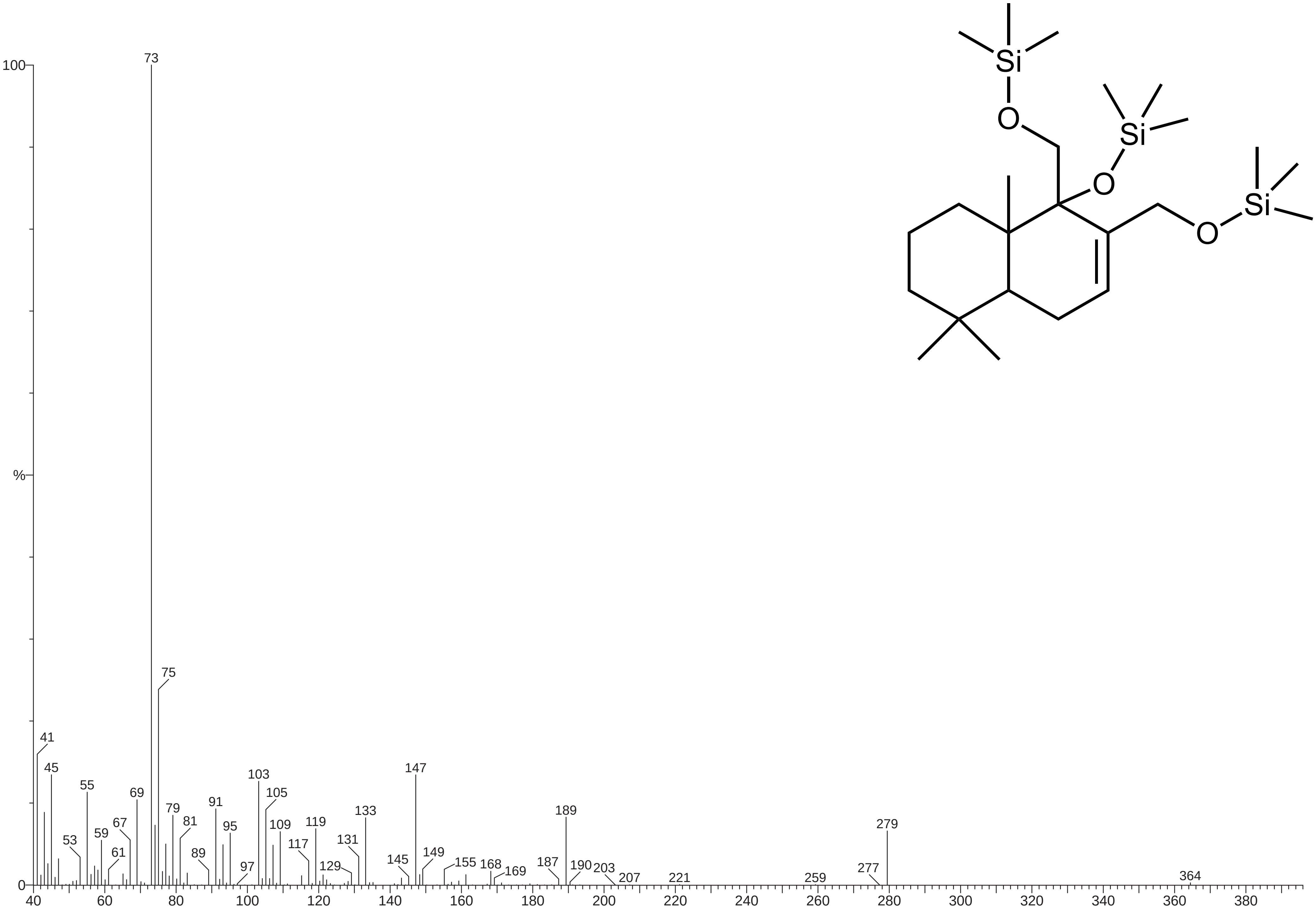
100
%
0
73
75
41
45
55
69
103
147
105
91
79
81
95
109
119
133
189
279
53
59
67
61
89
97
117
129
131
145
149
155
168
169
187
190
203
207
221
259
277
364
Si
O
40
60
80
100
120
140
160
180
200
220
240
260
280
300
320
340
360
380

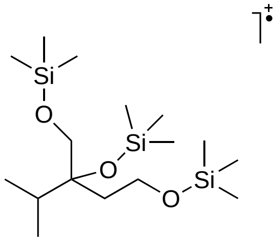

m/z = 364

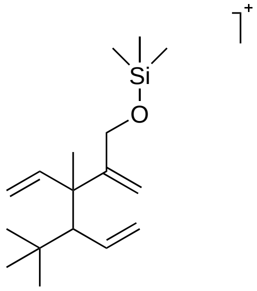

m/z = 279

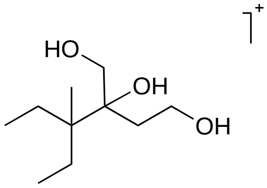

m/z = 189

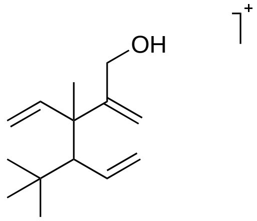

m/z = 207

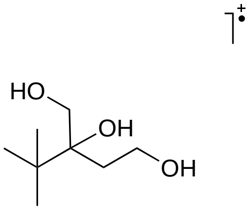

m/z = 162

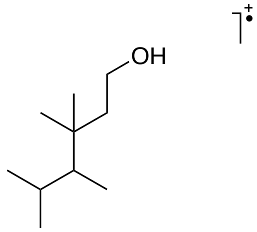

m/z = 158

Warburganal (**6**)

t_{Ret} = 45.1 min

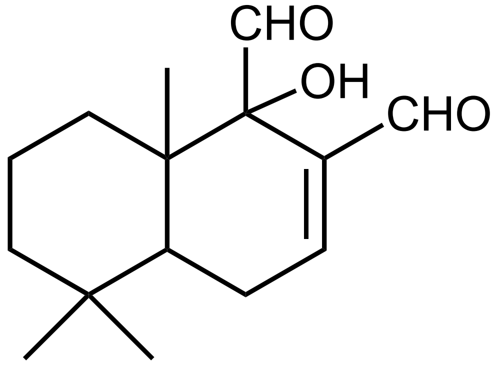

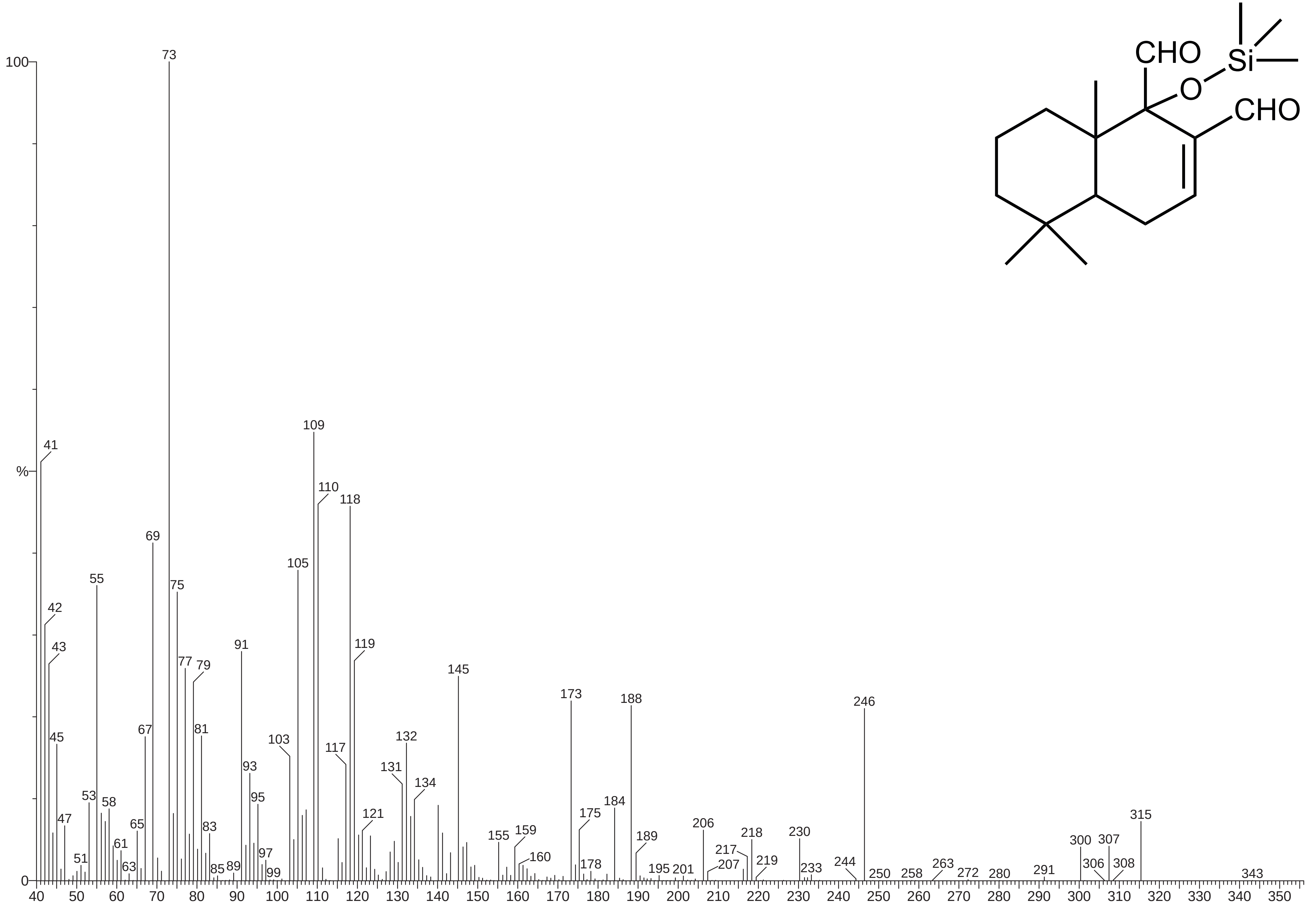
CHO
O
Si
CHO
100
%
0
73
109
41
110
118
69
105
55
75
42
43
91
119
77
79
145
173
188
246
45
67
81
103
132
117
93
131
134
95
53
58
47
121
184
175
315
65
83
155
159
206
189
218
230
300
307
61
97
217
160
51
63
85
89
99
178
207
219
244
195
201
233
263
306
308
291
272
280
250
258
343
40
50
60
70
80
90
100
110
120
130
140
150
160
170
180
190
200
210
220
230
240
250
260
270
280
290
300
310
320
330
340
350

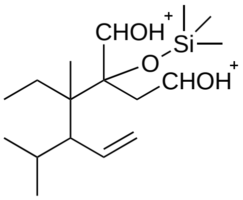

m/z = 315

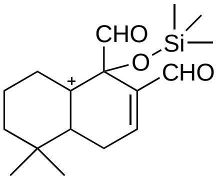

m/z = 307

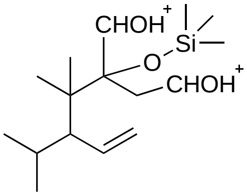

m/z = 300

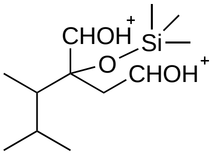

m/z = 246

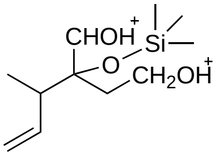

m/z = 230

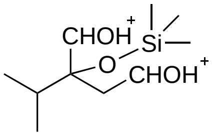

m/z = 218

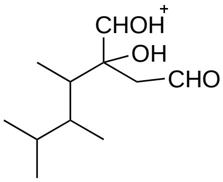

m/z = 201

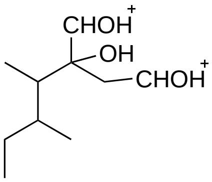

m/z = 188

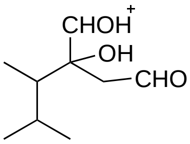

m/z = 173

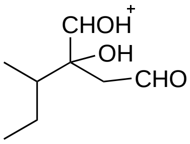

m/z = 159

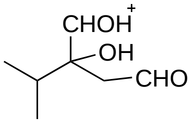

m/z = 145

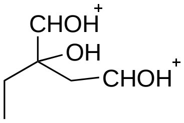

m/z = 132

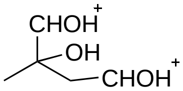

m/z = 118

Mukaadial (**7**)

t_{Ret} = 49.2 min

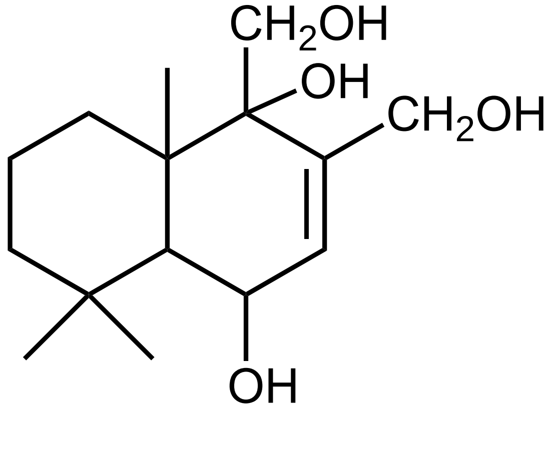

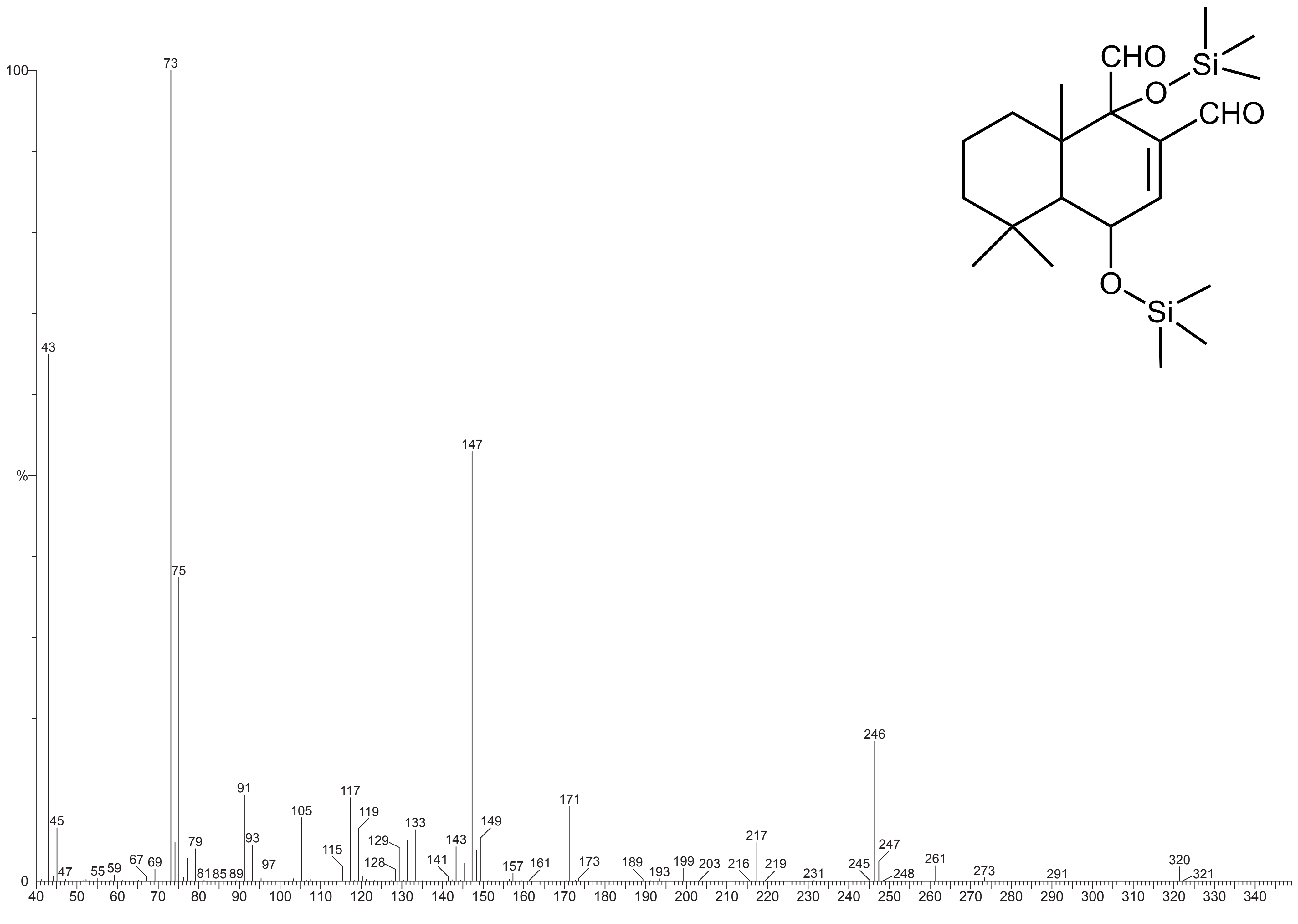
CHO
O
Si
CHO
O
Si
100
%
0
73
43
45
47
55
59
67
69
75
79
81
85
89
91
93
97
105
115
117
119
128
129
133
141
143
147
149
157
161
171
173
189
193
199
203
216
217
219
231
245
246
247
248
261
273
291
320
321
40
50
60
70
80
90
100
110
120
130
140
150
160
170
180
190
200
210
220
230
240
250
260
270
280
290
300
310
320
330
340

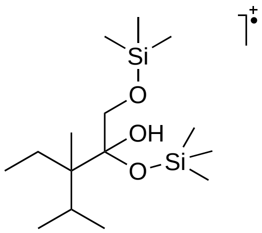

m/z = 320

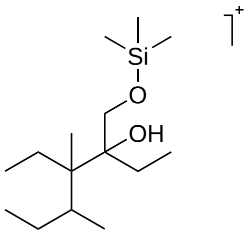

m/z = 273

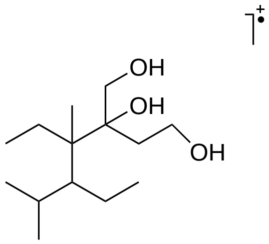

m/z = 246

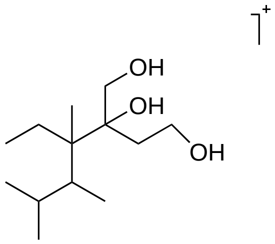

m/z = 231

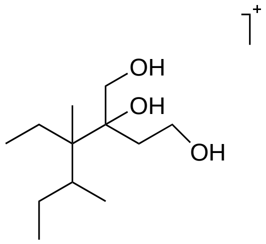

m/z = 217

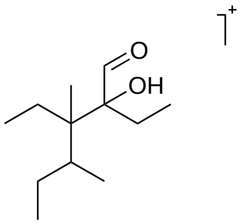

m/z = 199

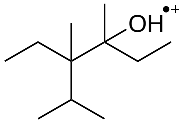

m/z = 171

8

t_{Ret} = 46.3 min

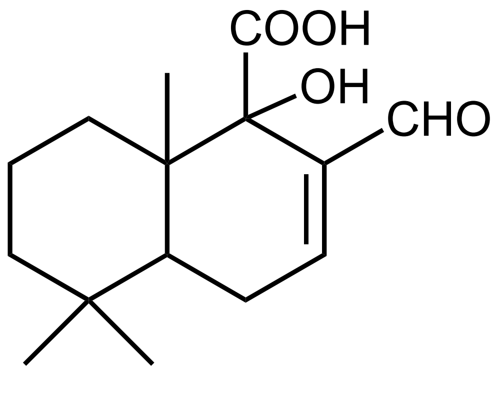

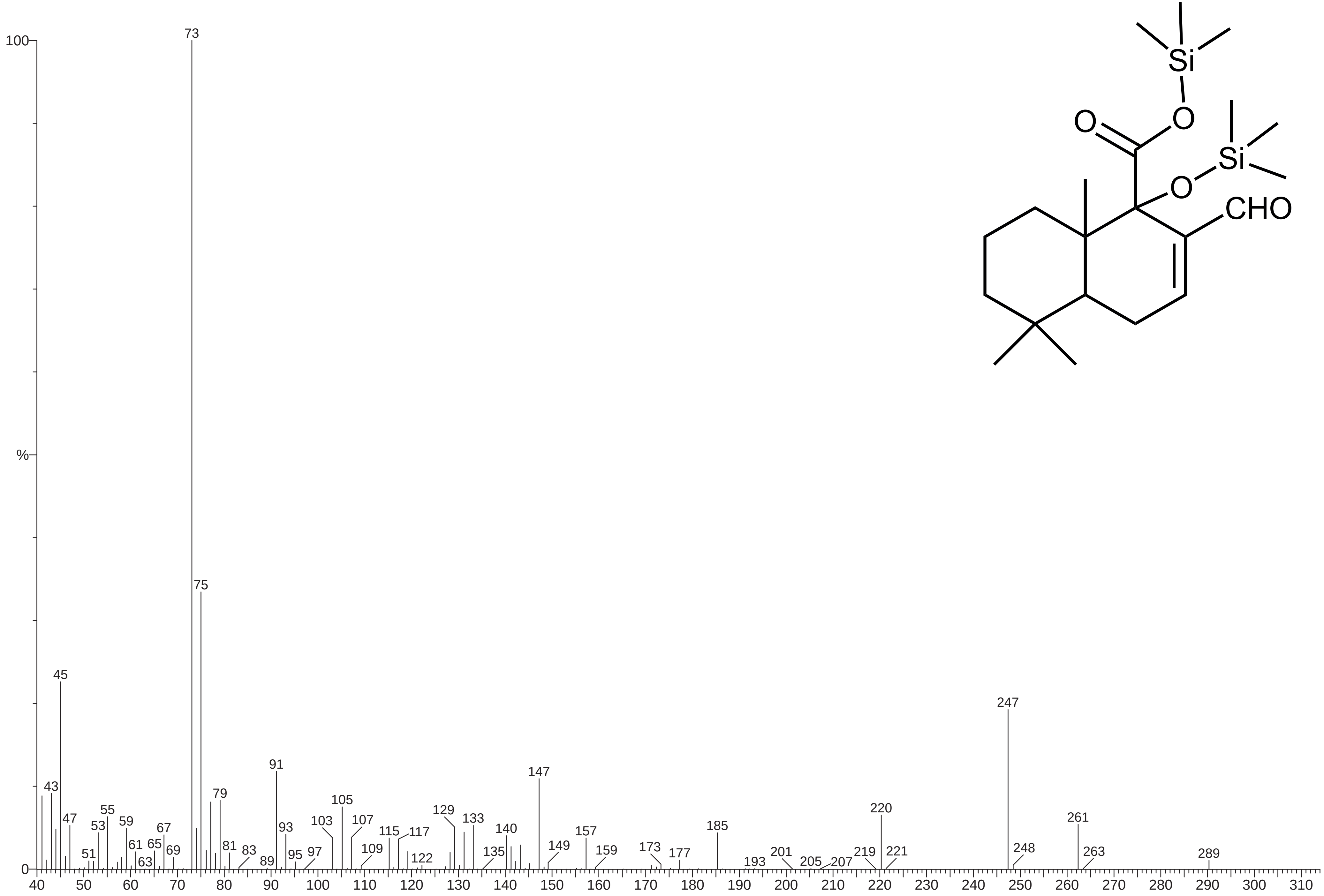
100
%
0
73
75
45
43
47
51
53
55
59
61
63
65
67
69
79
81
83
89
91
93
95
97
103
105
107
109
115
117
122
129
133
135
140
147
149
157
159
173
177
185
193
201
205
207
219
220
221
247
248
261
263
289
40
50
60
70
80
90
100
110
120
130
140
150
160
170
180
190
200
210
220
230
240
250
260
270
280
290
300
310
Si
O
O
O
Si
CHO

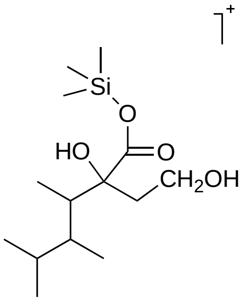

m/z = 289

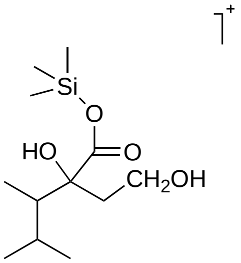

m/z = 261

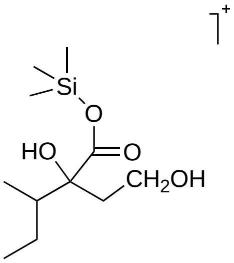

m/z = 247

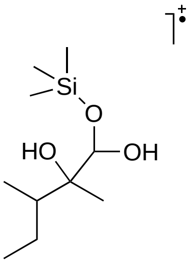

m/z = 220

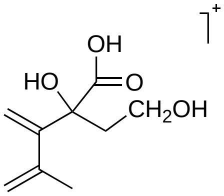

m/z = 185

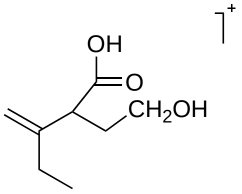

m/z = 157

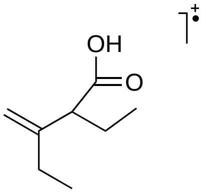

m/z = 142

Pereniporin A (**9**)

t_{Ret} = 46.9 min

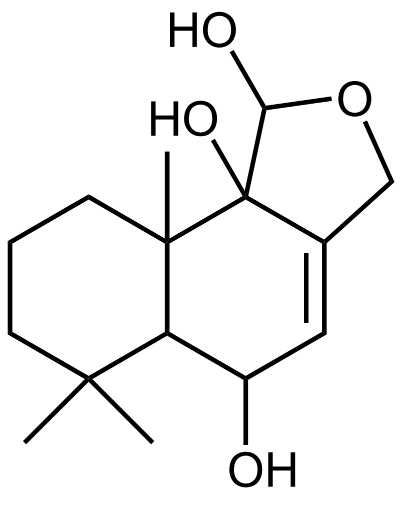

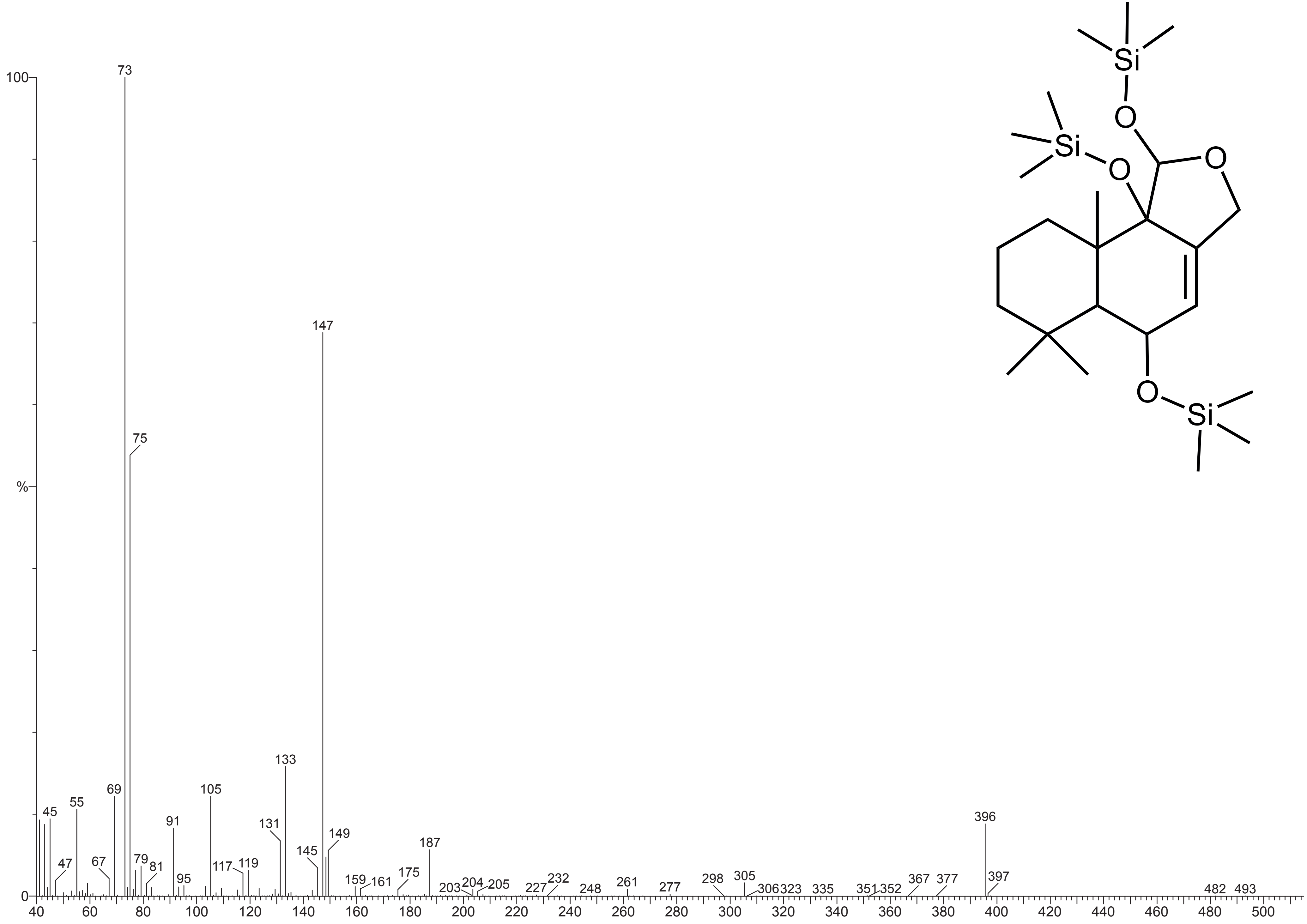

100
%
0
73
147
75
133
69
105
55
45
91
131
149
187
145
47
67
79
81
95
117
119
159
161
175
203
204
205
227
232
248
261
277
298
305
306
323
335
351
352
367
377
396
397
482
493
40 60 80 100 120 140 160 180 200 220 240 260 280 300 320 340 360 380 400 420 440 460 480 500
Si
O
Si
O
O
O
Si

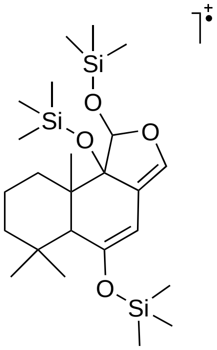

m/z = 482

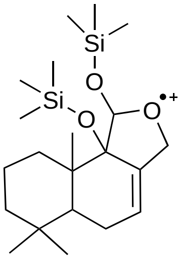

m/z = 396

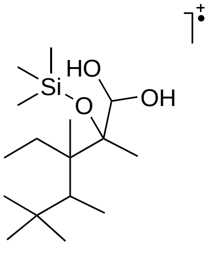

m/z = 305

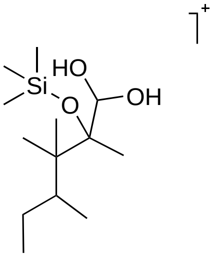

m/z = 261

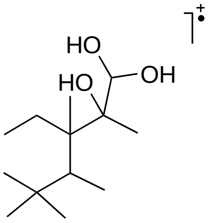

m/z = 232

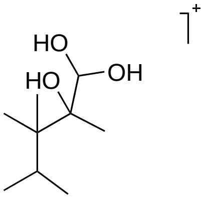

m/z = 175

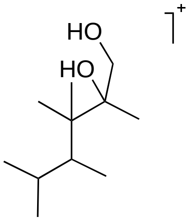

m/z = 187

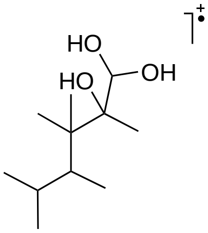

m/z = 204

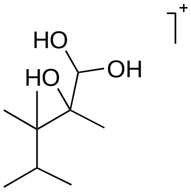

m/z = 175

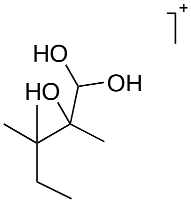

m/z = 161

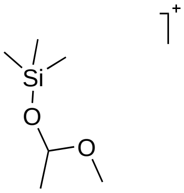

m/z = 147

10

t_{Ret} = 47.6 min

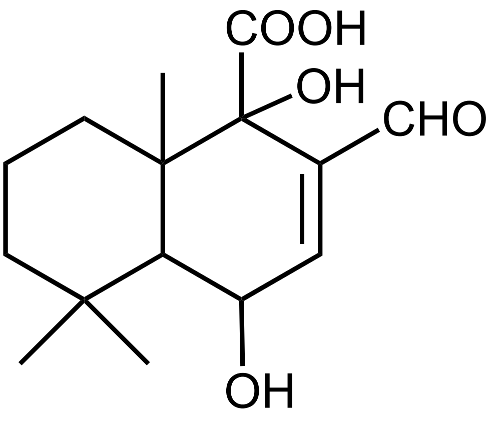

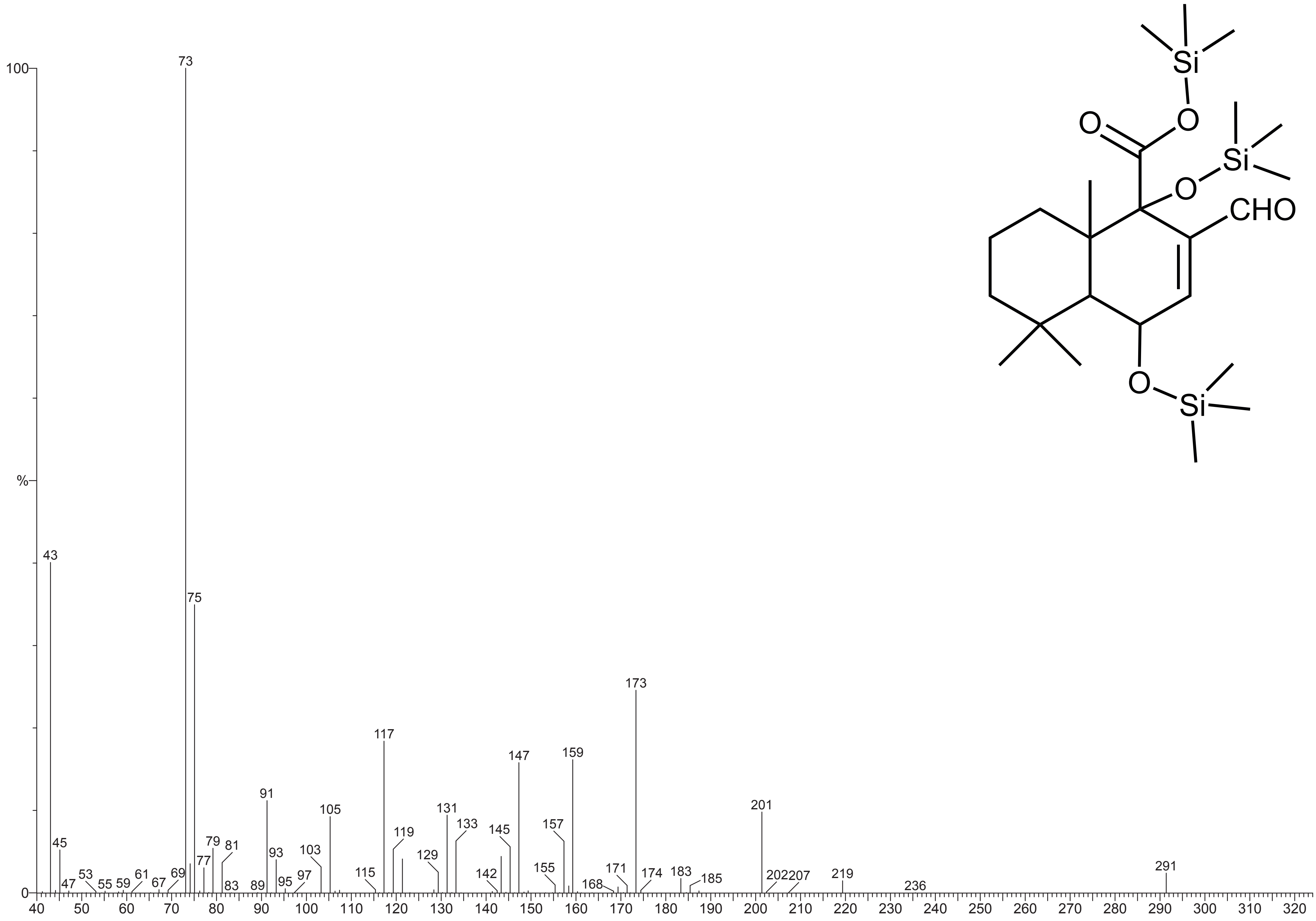

100
%
0
73
43
75
173
117
147
159
91
105
201
131
133
145
157
119
45
79
81
93
103
129
77
115
142
155
171
174
183
185
202
207
219
291
236
47
53
55
59
61
67
69
83
89
95
97
168
40
50
60
70
80
90
100
110
120
130
140
150
160
170
180
190
200
210
220
230
240
250
260
270
280
290
300
310
320
Si
O
O
Si
O
CHO
O
Si

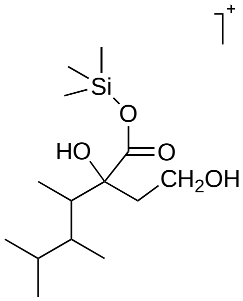

m/z = 289

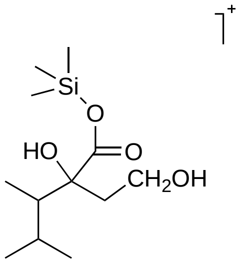

m/z = 261

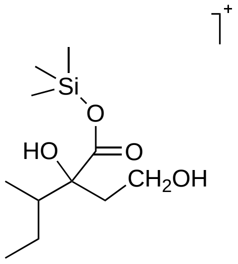

m/z = 247

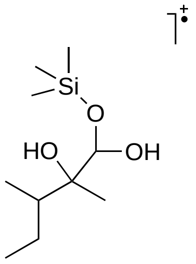

m/z = 220

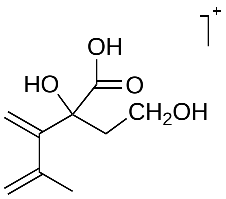

m/z = 185

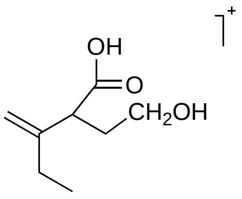

m/z = 157

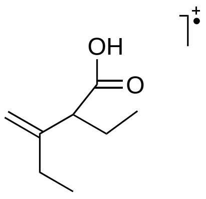

m/z = 142

11

t_{Ret} = 47.6 min

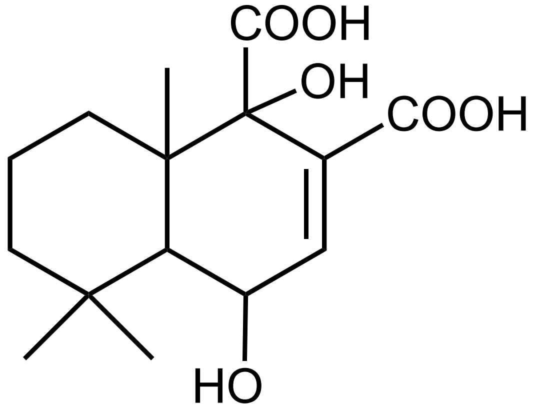

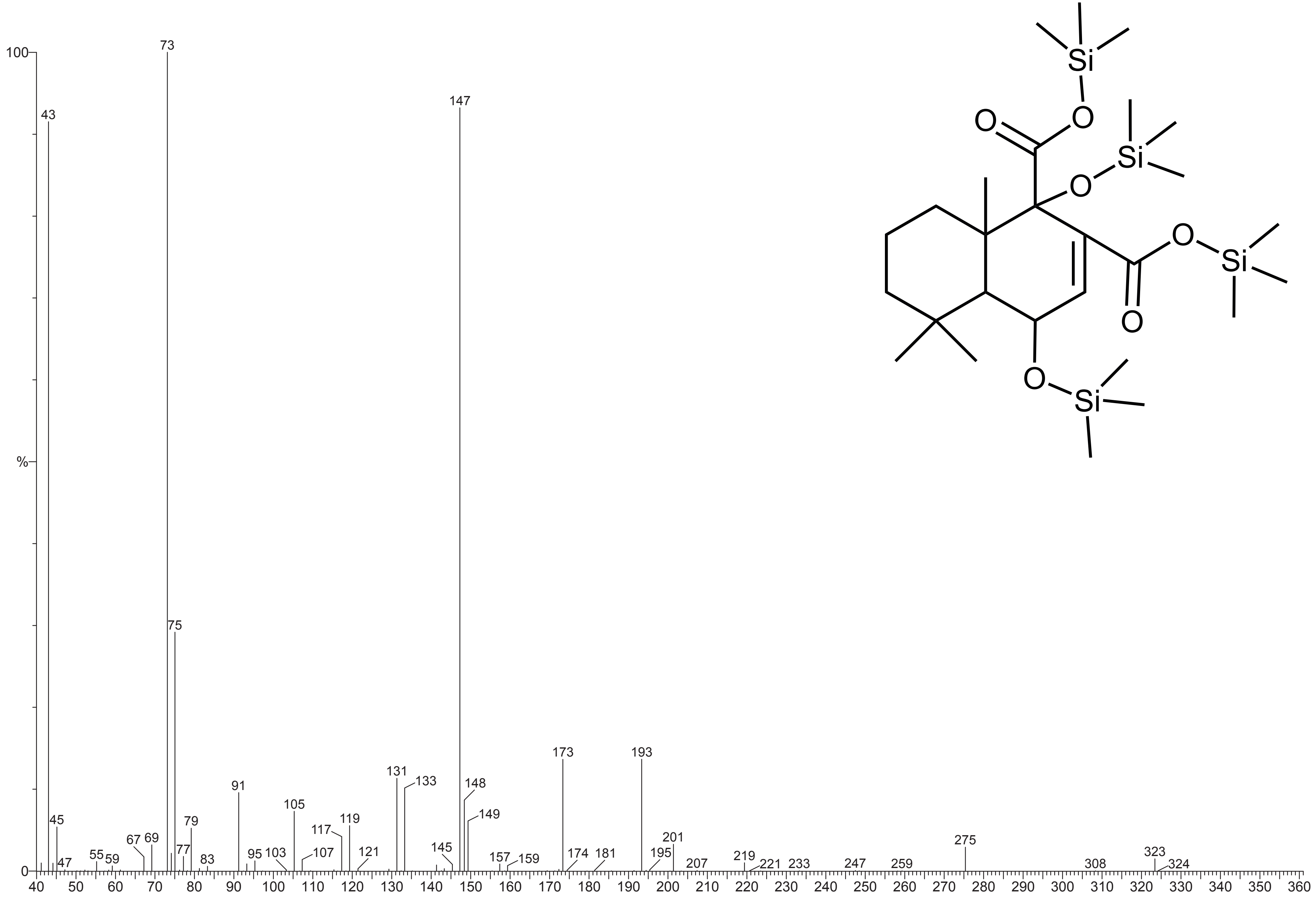

100
%
0
73
147
43
75
45
47
55
59
67
69
77
79
83
91
95
103
105
107
117
119
121
131
133
145
148
149
157
159
173
174
181
193
195
201
207
219
221
233
247
259
275
308
323
324
40 50 60 70 80 90 100 110 120 130 140 150 160 170 180 190 200 210 220 230 240 250 260 270 280 290 300 310 320 330 340 350 360
O
Si

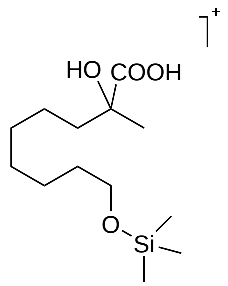

m/z = 275

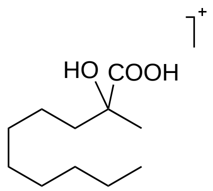

m/z = 201

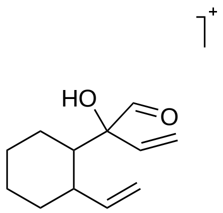

m/z = 193

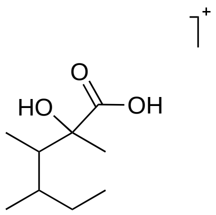

m/z = 173

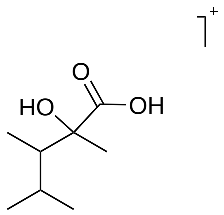

m/z = 159

12-Hydroxy-*epi*-albrassitriol
(**12**)

t_{Ret} = 46.2 min

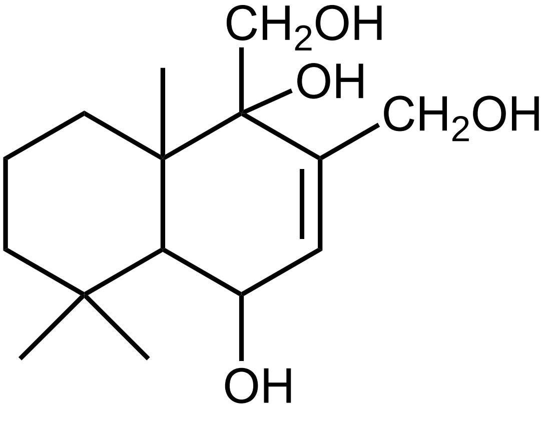

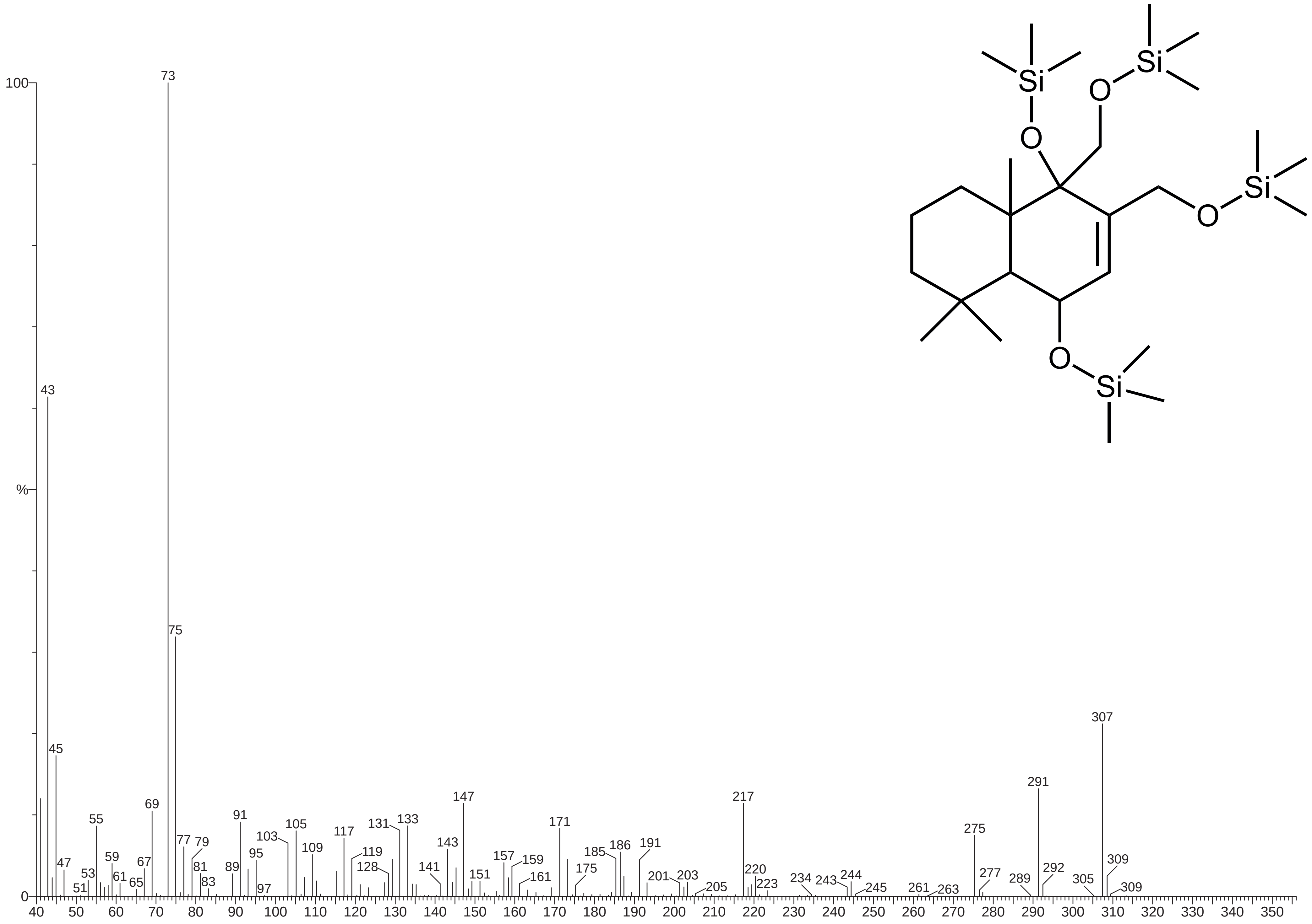

100
%
0
40 50 60 70 80 90 100 110 120 130 140 150 160 170 180 190 200 210 220 230 240 250 260 270 280 290 300 310 320 330 340 350
43 45 47 51 53 55 59 61 65 67 69 73 75 77 79 81 83 89 91 95 97 103 105 109 117 119 128 131 133 141 143 147 151 157 159 161 171 175 185 186 191 201 203 205 217 220 223 234 243 244 245 261 263 275 277 289 291 292 305 307 309 309
Si
O

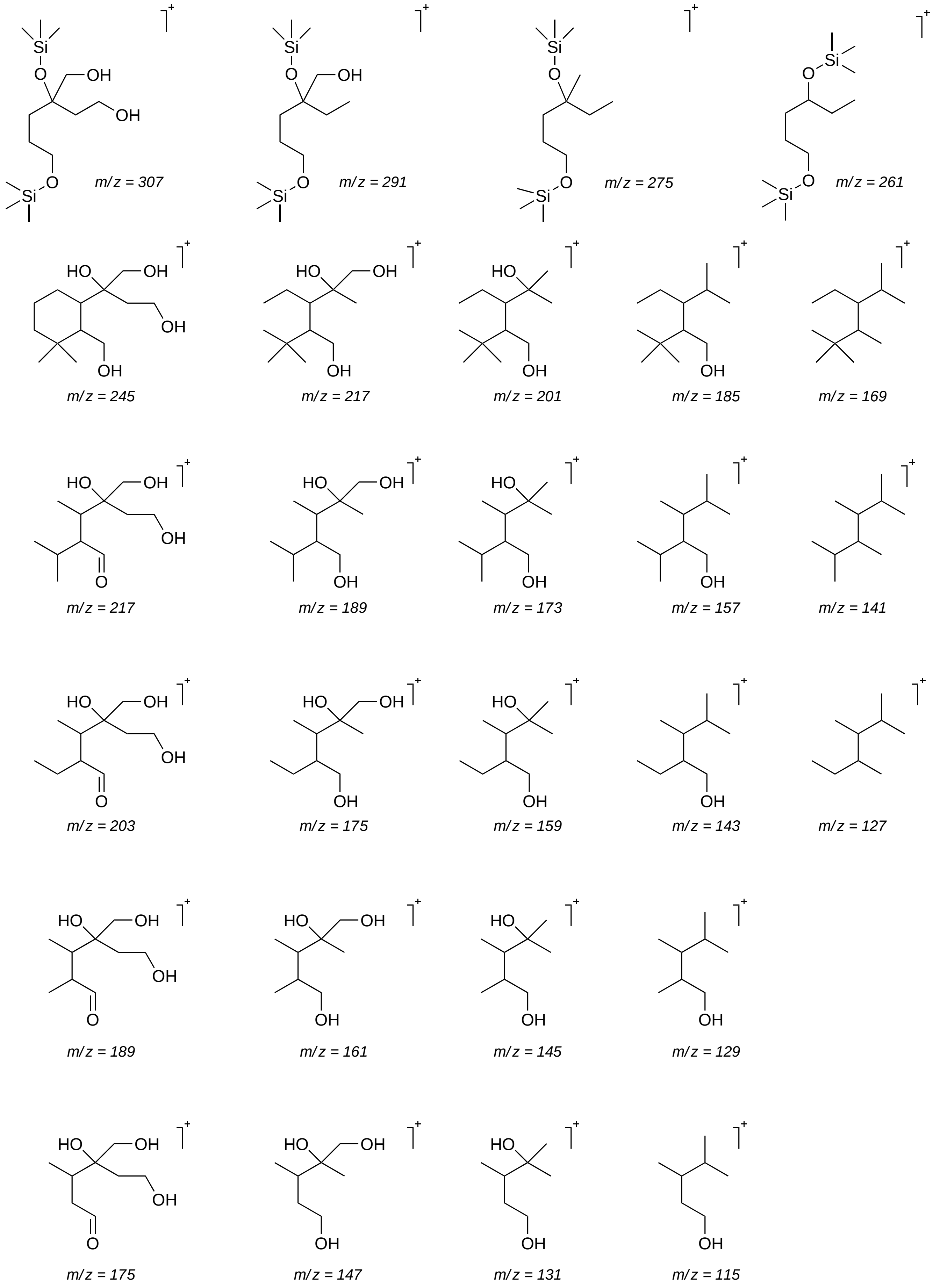
m/z = 307
m/z = 291
m/z = 275
m/z = 261
m/z = 245
m/z = 217
m/z = 201
m/z = 185
m/z = 169
m/z = 217
m/z = 189
m/z = 173
m/z = 157
m/z = 141
m/z = 203
m/z = 175
m/z = 159
m/z = 143
m/z = 127
m/z = 189
m/z = 161
m/z = 145
m/z = 129
m/z = 175
m/z = 147
m/z = 131
m/z = 115

13

t_{Ret} = 45.5 min

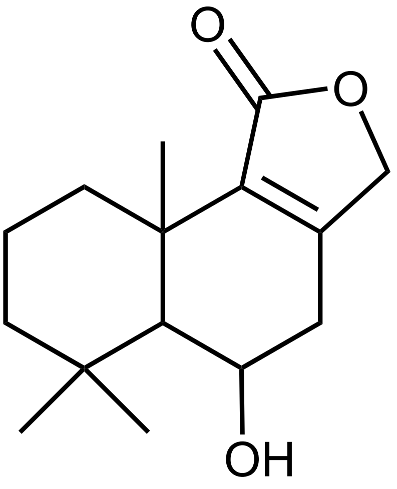

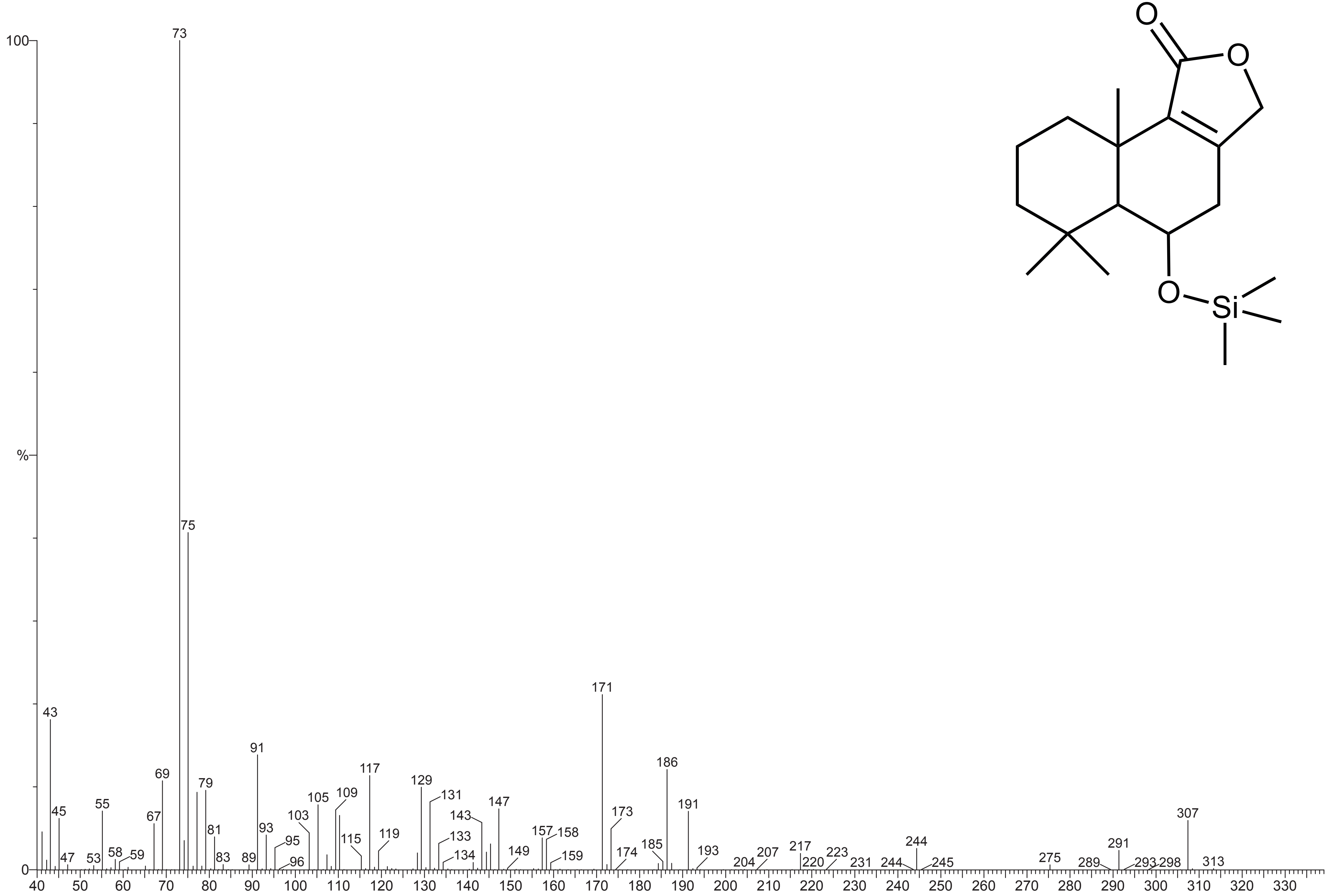
100
%
0
73
75
43
45
47
53
55
58
59
67
69
79
81
83
89
91
93
95
96
103
105
109
115
117
119
129
131
133
134
143
147
149
157
158
159
171
173
174
185
186
191
193
204
207
217
220
223
231
244
244
245
275
289
291
293
298
307
313
40 50 60 70 80 90 100 110 120 130 140 150 160 170 180 190 200 210 220 230 240 250 260 270 280 290 300 310 320 330
O
O
O
Si

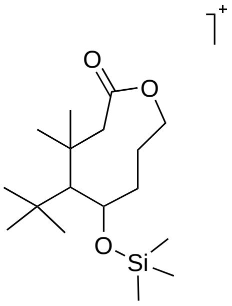

m/z = 313

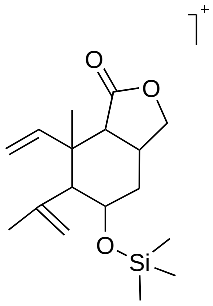

m/z = 307

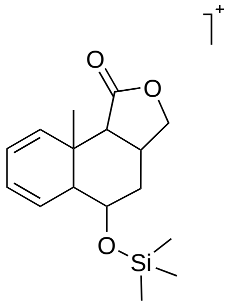

m/z = 292

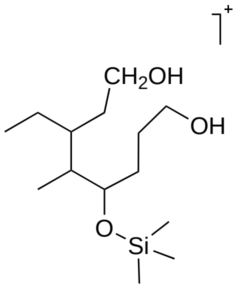

m/z = 275

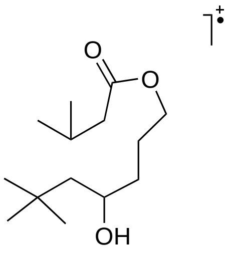

m/z = 244

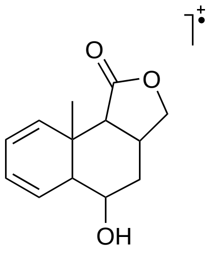

m/z = 220

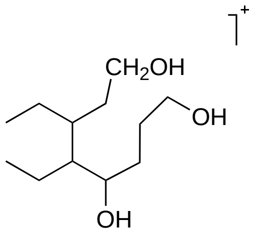

m/z = 217

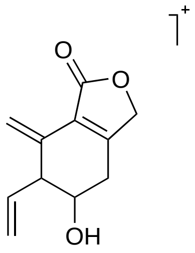

m/z = 191

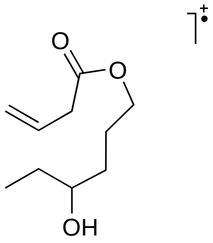

m/z = 186

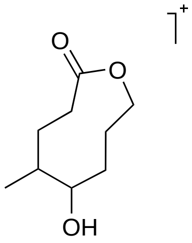

m/z = 171

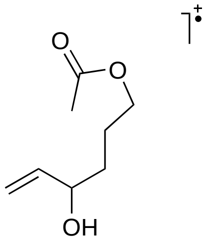

m/z = 158

14

t_{Ret} = 48.1 min

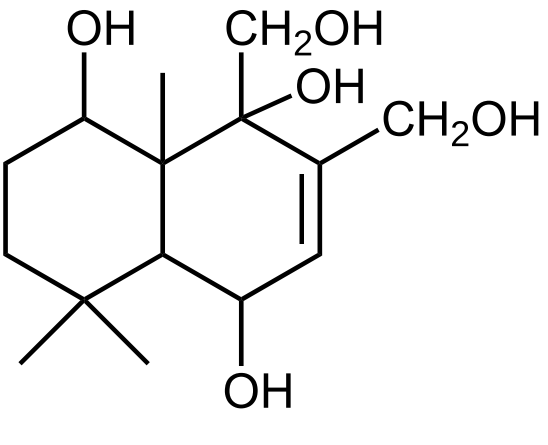

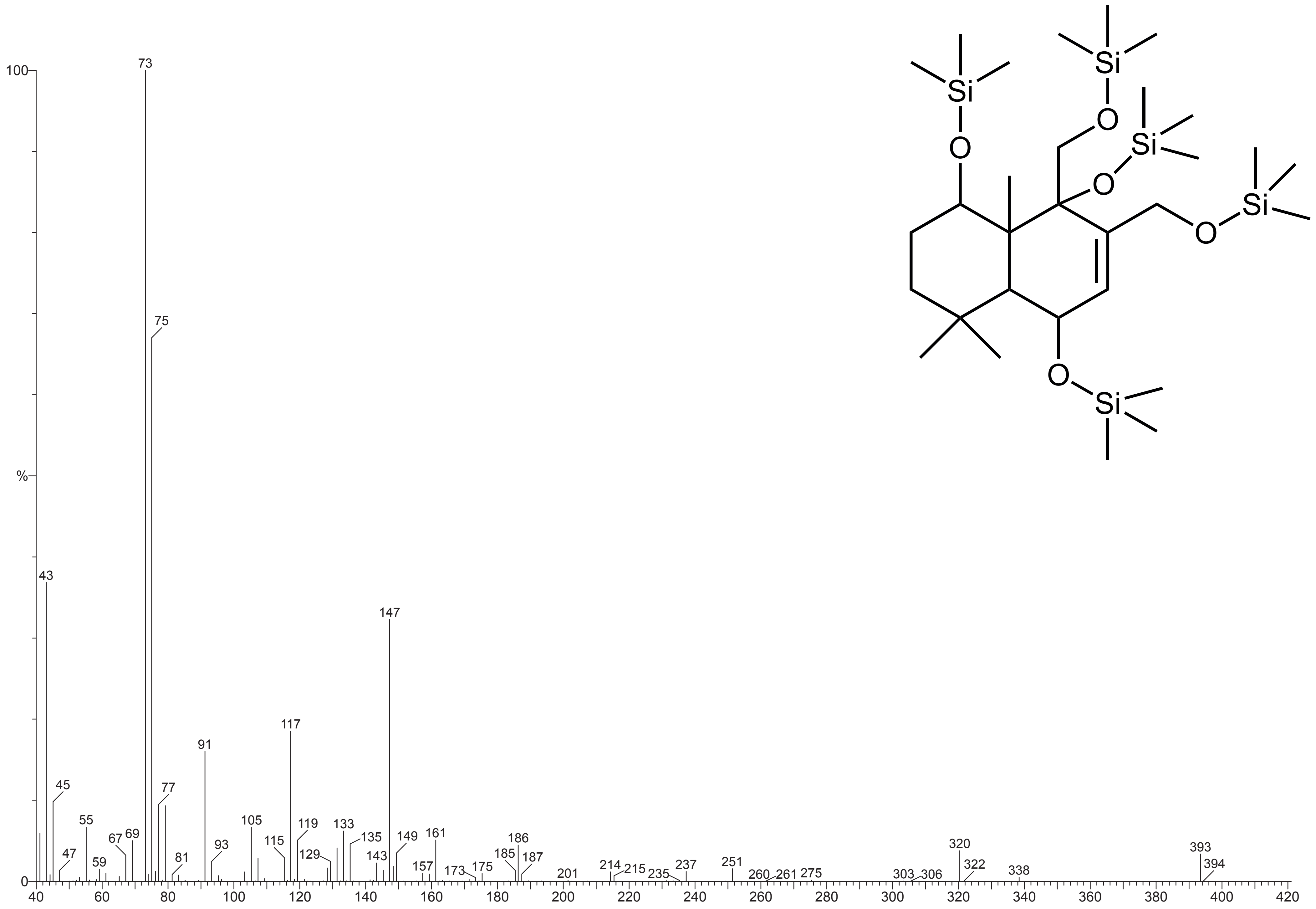

100
%
0
73
75
43
147
117
91
45
77
55
105
119
133
67
69
135
149
161
186
93
115
129
47
59
81
143
157
173
175
185
187
320
393
394
201
214
215
235
237
251
260
261
275
303
306
322
338
40
60
80
100
120
140
160
180
200
220
240
260
280
300
320
340
360
380
400
420
Si
O

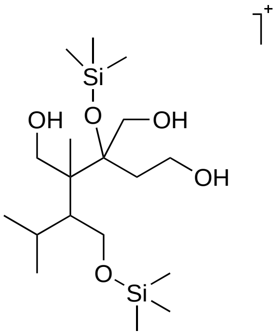

m/z = 393

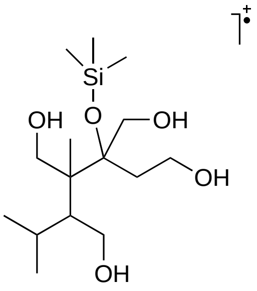

m/z = 322

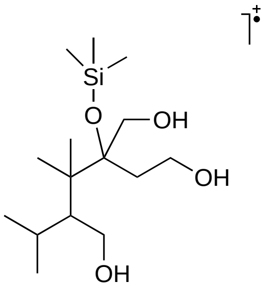

m/z = 306

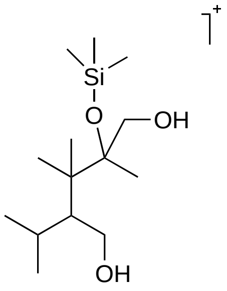

m/z = 275

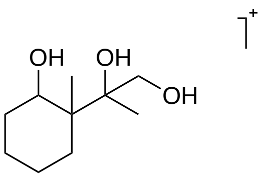

m/z = 187

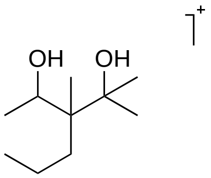

m/z = 173

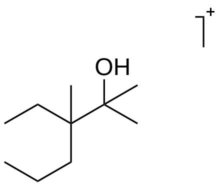

m/z = 157

15

t_{Ret} = 49.8 min

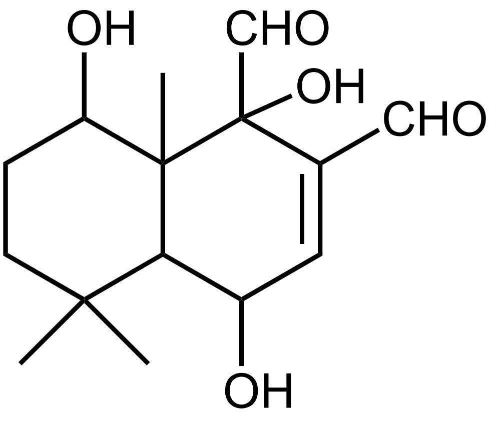

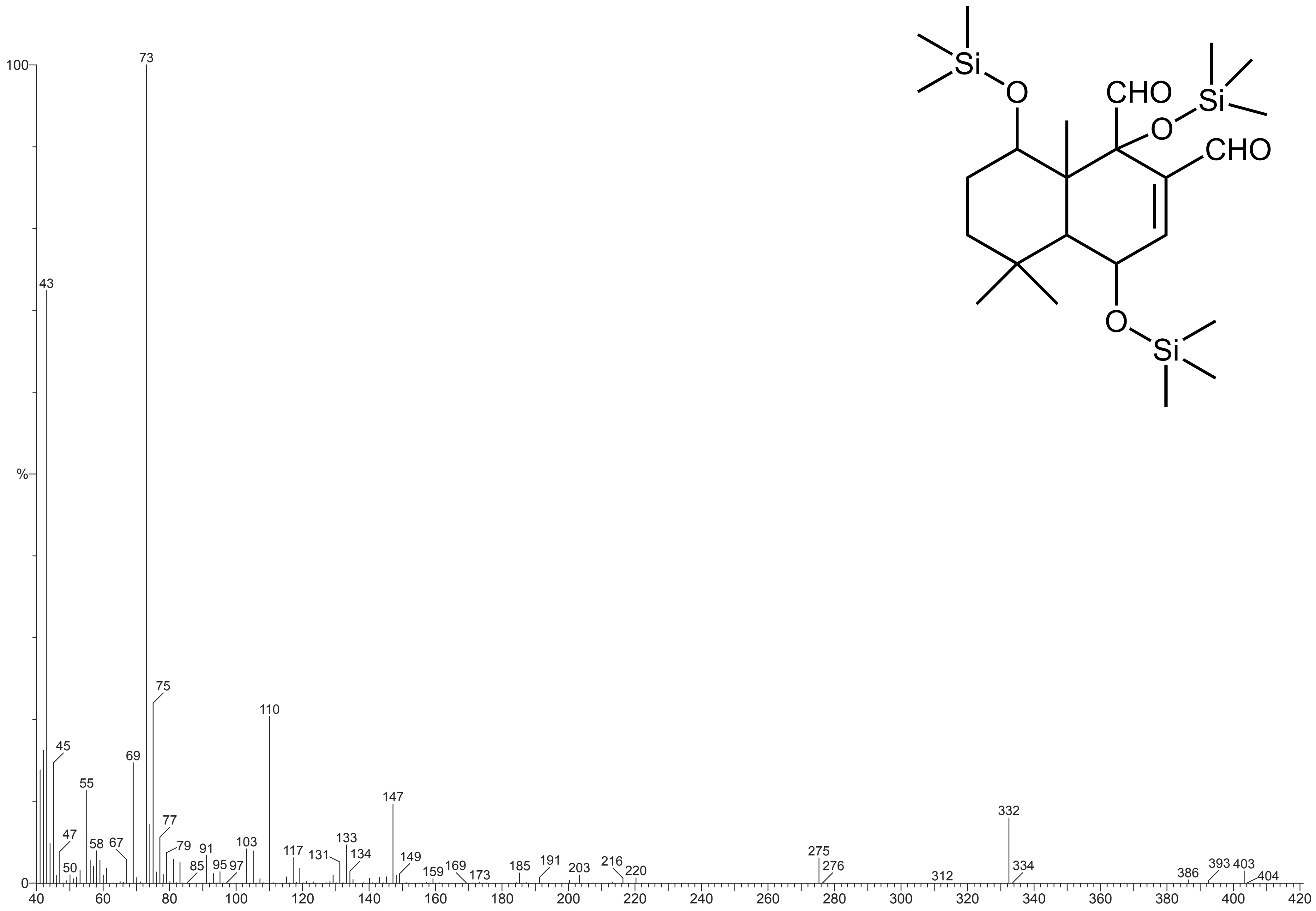

100
%
0
73
43
45
69
55
47
58
67
50
75
77
79
85
91
95
97
103
110
117
131
133
134
147
149
159
169
173
185
191
203
216
220
275
276
312
332
334
386
393
403
404
40
60
80
100
120
140
160
180
200
220
240
260
280
300
320
340
360
380
400
420
Si
O
CHO
Si
O
CHO
O
Si

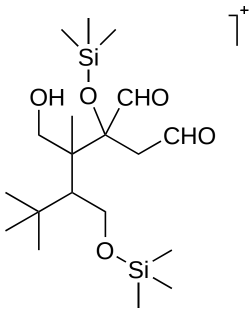

m/z = 403

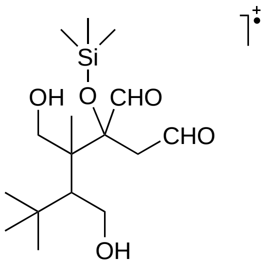

m/z = 332

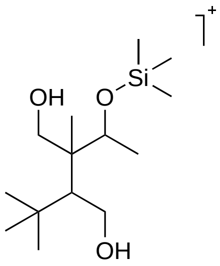

m/z = 275

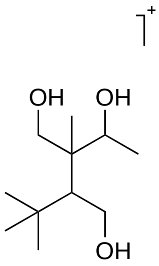

m/z = 203

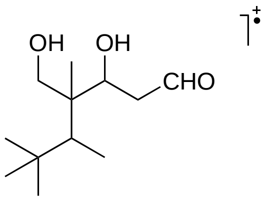

m/z = 216

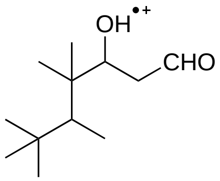

m/z = 200

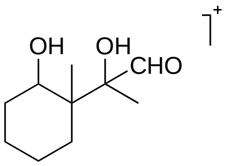

m/z = 185

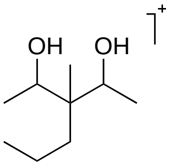

m/z = 159

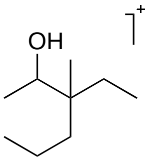

m/z = 143

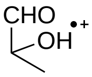

m/z = 74

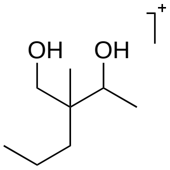

m/z = 145

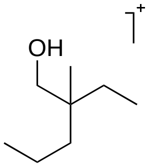

m/z = 129

Deacetylugandensiolide
(**16**)

t_{Ret} = 46.6 min

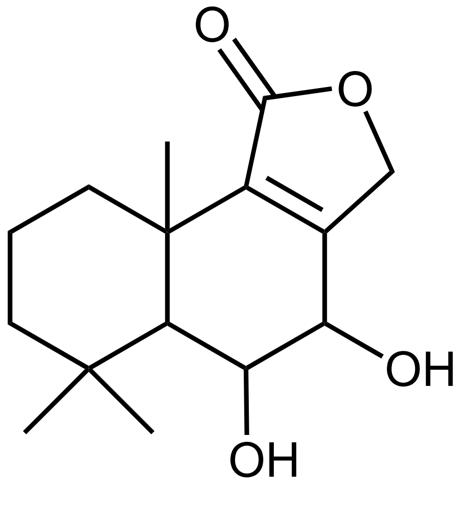

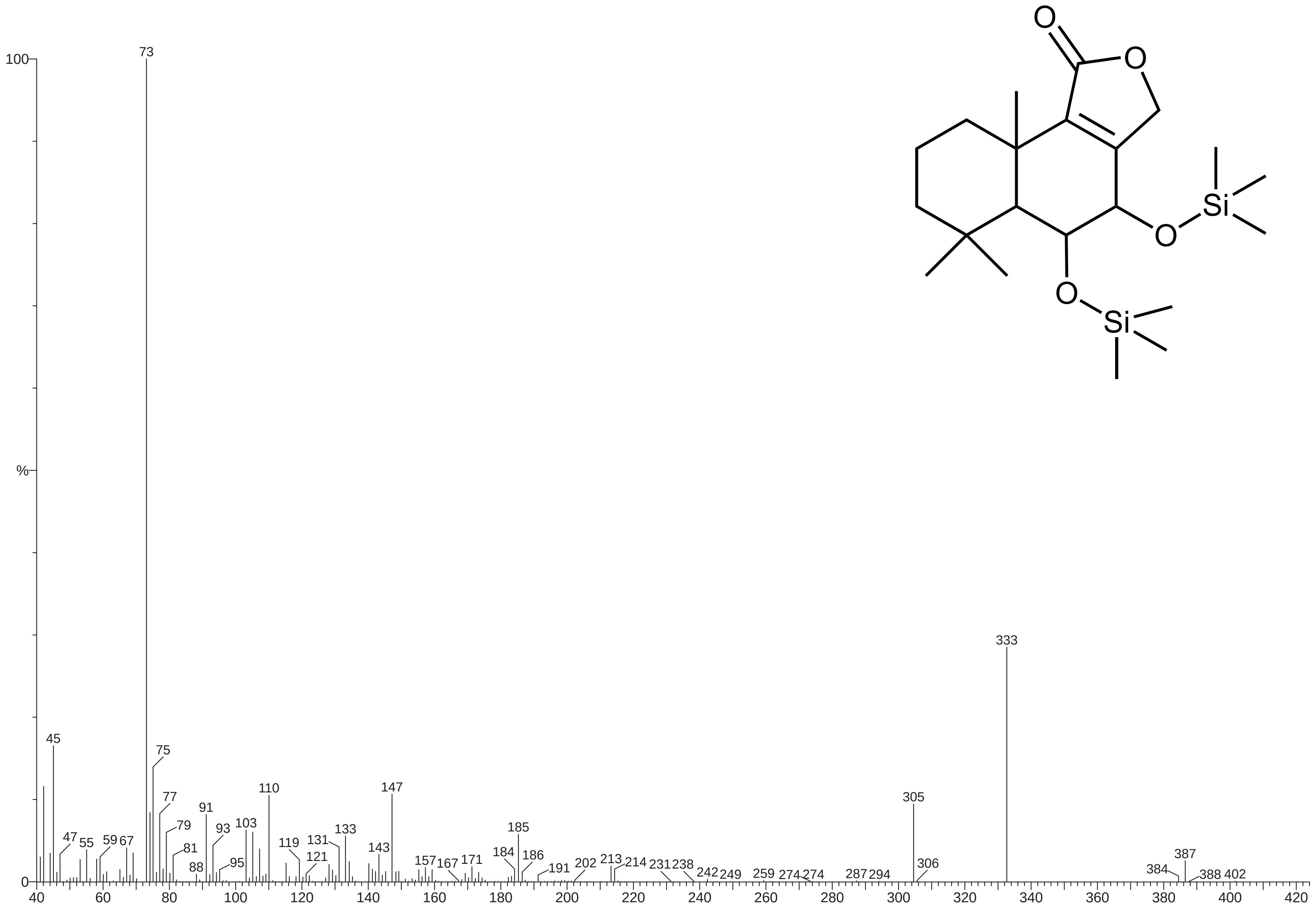
100
%
0
73
45
75
77
79
81
88
91
93
95
103
110
119
121
131
133
143
147
157
167
171
184
185
186
191
202
213
214
231
238
242
249
259
274
274
287
294
305
306
333
384
387
388
402
40
60
80
100
120
140
160
180
200
220
240
260
280
300
320
340
360
380
400
420
O
O
O
O
Si
Si

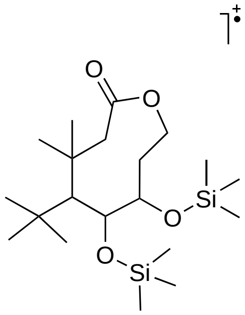

m/z = 402

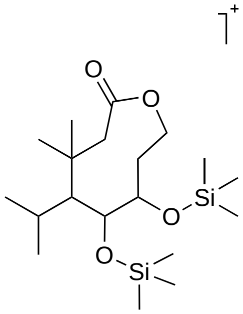

m/z = 387

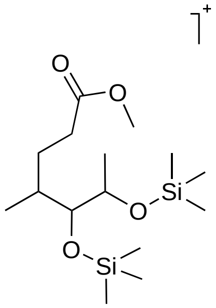

m/z = 333

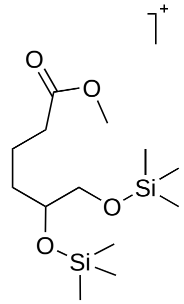

m/z = 305

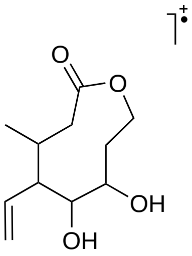

m/z = 214

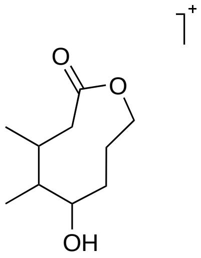

m/z = 185

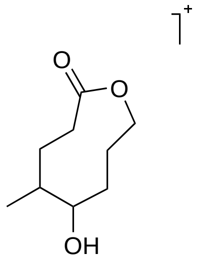

m/z = 171

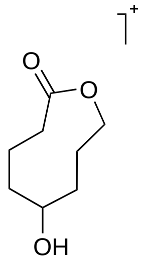

m/z = 157

17

t_{Ret} = 50.6 min

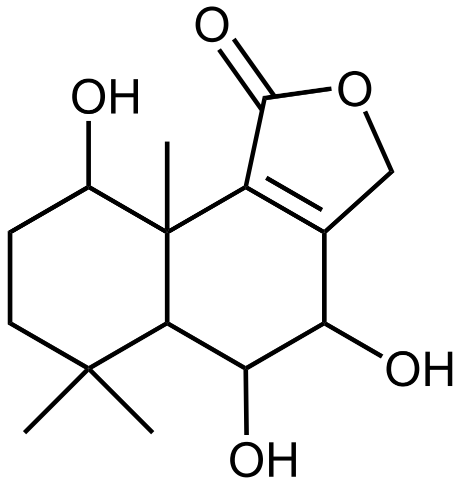

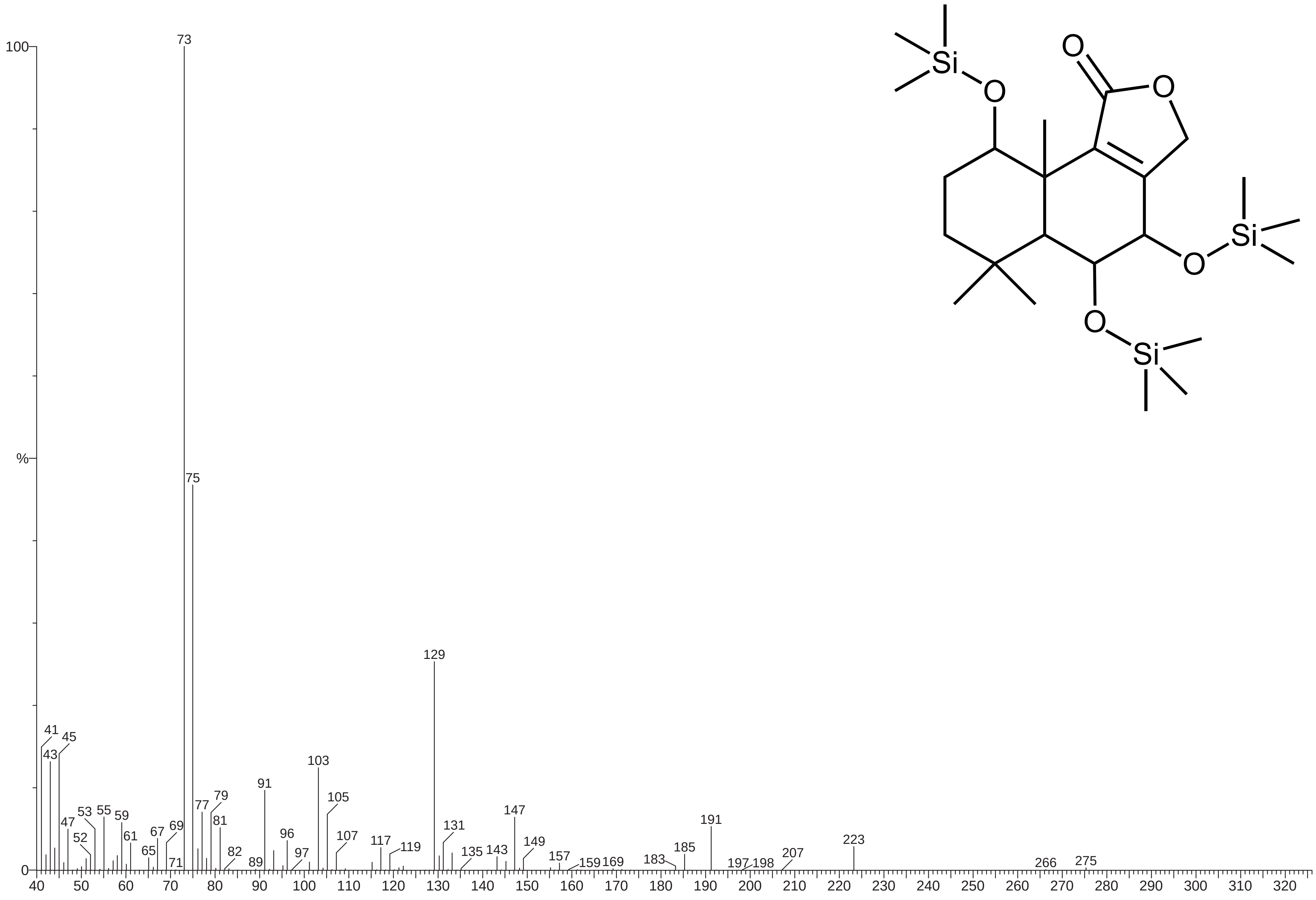

100
%
0
73
75
129
41
43
45
47
52
53
55
59
61
65
67
69
71
77
79
81
82
89
91
96
97
103
105
107
117
119
131
135
143
147
149
157
159
169
183
185
191
197
198
207
223
266
275
40 50 60 70 80 90 100 110 120 130 140 150 160 170 180 190 200 210 220 230 240 250 260 270 280 290 300 310 320
Si
O
O
O
O
Si
O
O
Si

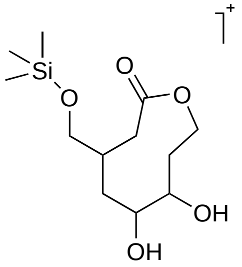

m/z = 275

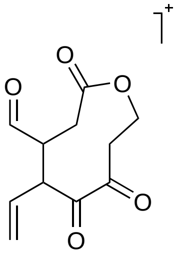

m/z = 224

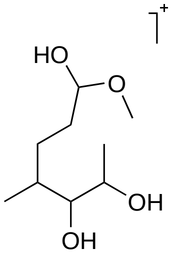

m/z = 191

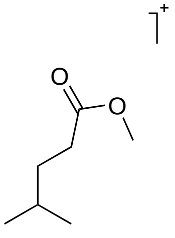

m/z = 129

18

t_{Ret} = 63.9 min

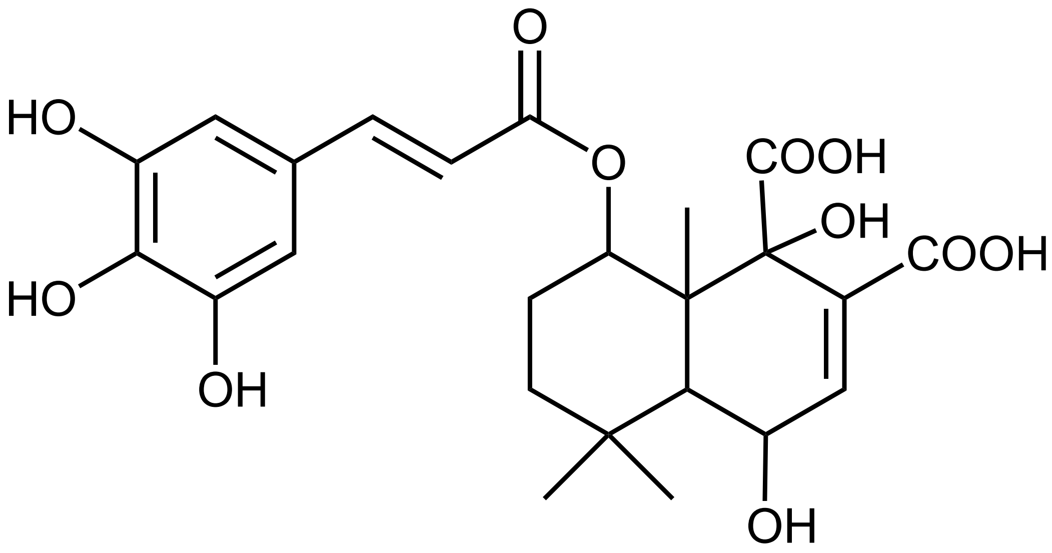

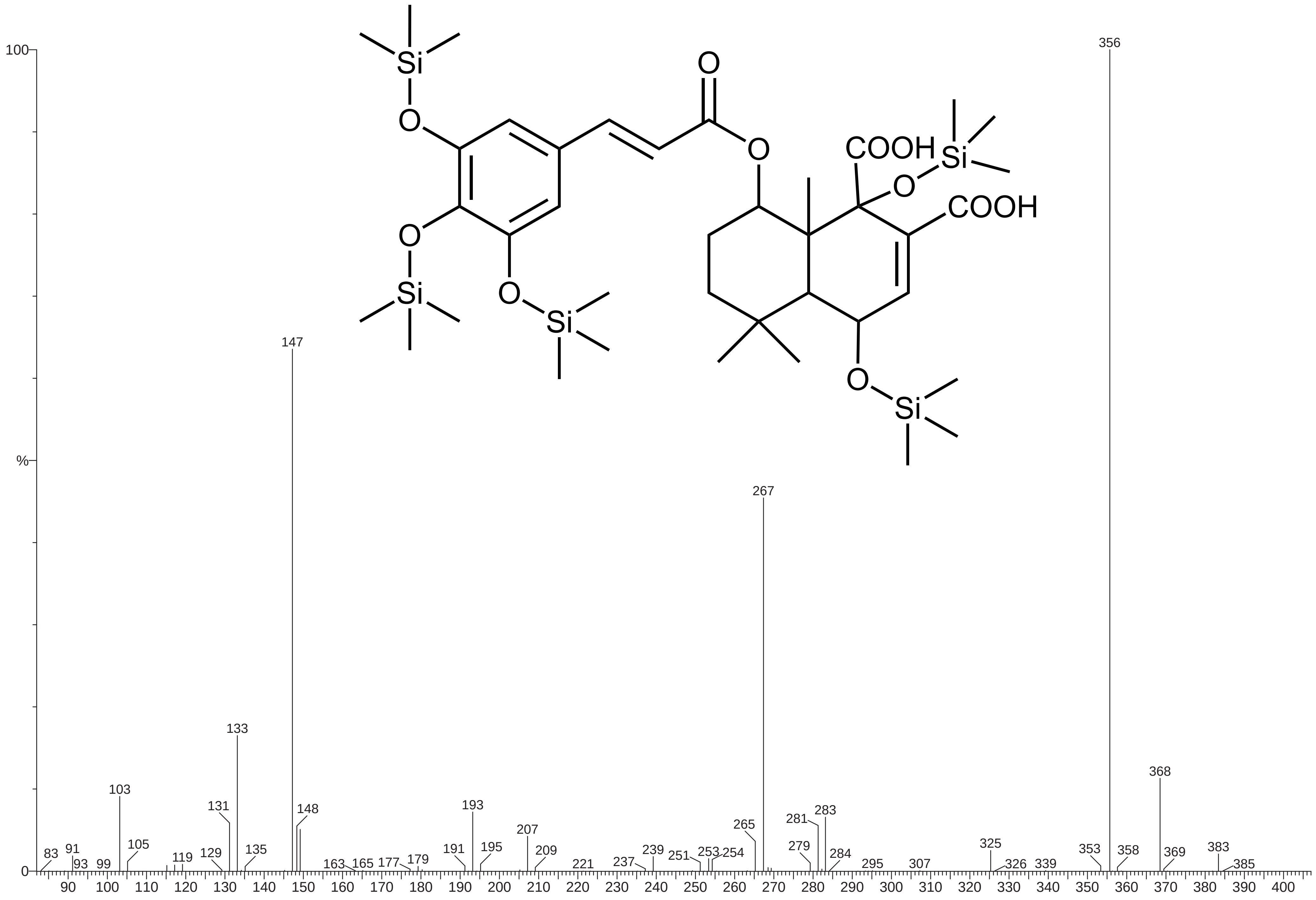

100
%
0
90 100 110 120 130 140 150 160 170 180 190 200 210 220 230 240 250 260 270 280 290 300 310 320 330 340 350 360 370 380 390 400
356
147
267
133
368
103
131
148
193
207
265
281
283
83
91
93
99
105
119
129
135
163
165
177
179
191
195
209
221
237
239
251
253
254
279
284
295
307
325
326
339
353
358
369
383
385
Si
O
O
O
Si
O
Si
O
COOH
COOH
O
Si
O
Si

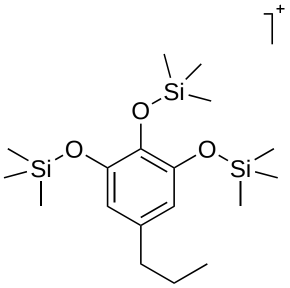

m/z = 383

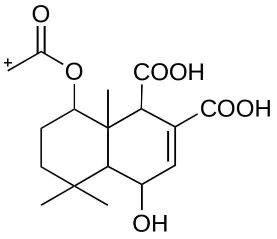

m/z = 339

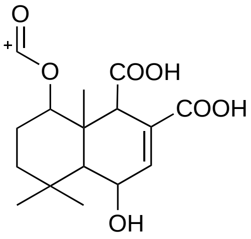

m/z = 325

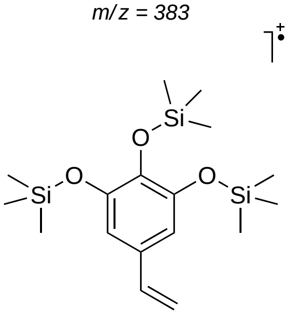

m/z = 368

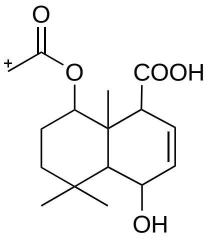

m/z = 295

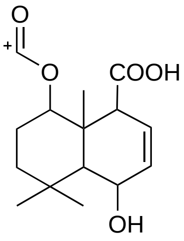

m/z = 281

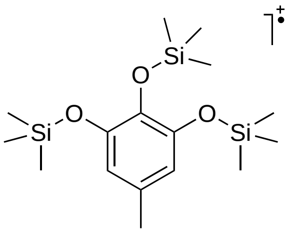

m/z = 356

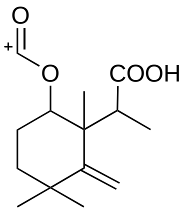

m/z = 267

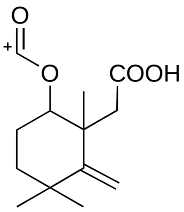

m/z = 239

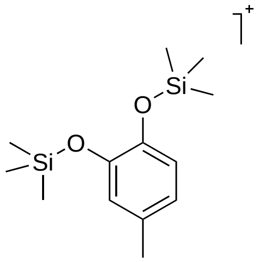

m/z = 267

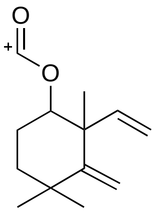

m/z = 207

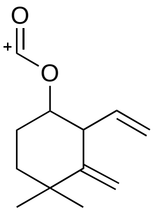

m/z = 193

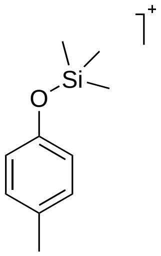

m/z = 179
