# Supplementary material for: Antimicrobial drimane sesquiterpenes and their effect on endophyte communities in the medical tree Warburgia ugandensis
Source: Front Microbiol. 2014 Feb 7;5:13. doi: 10.3389/fmicb.2014.00013 (PMC3916764; doi:10.3389/fmicb.2014.00013)
Supplement: Supplementary Data S1 — Tentative Structures of all Drimane Sesquiterpene Analytes from Warburgia ugandensis. This file provides information on which the tentative structure identification of drimane sesquiterpenes analytes is based in this study. Each analyte is presented on three pages: Page 1 contains the tentative structure with the analysis retention time, page 2 presents the MS spectrum together with the structure of the derivatized analyte, and page 3 illustrates structure fragments corresponding to specific fragments in the EI–MS spectrum. The tentative structure assignment is based on these data for each analyte respectively. The numbering corresponds to that presented in Figure 2. [file Presentation1.ZIP › Table S2.pdf]

Table S2. Bacterial endophytes from leaves, fruits and roots of *Warburgia ugandensis*. Sequence analysis of a partial 16S rDNA (approximately 500 bp at the 5´end) clone library prepared from isolated bacterial DNA.

Clone ID	Number of clones	Closest identified relative (blastn)		
		ID	Accession No.	Similarity
Actinobacteria				
39	2	*Curtobacterium flaccumfaciens*	AM410688	502/504 (99%)
50	1	*Curtobacterium flaccumfaciens*	AM410688	417/444 (93%)
41	1	*Nocardiaceae bacterium*	AM921641	495/499 (99%)
Firmicutes				
32	1	*Bacillus benzoevorans*	DQ333291	529/531 (99%)
25	1	*Bacillus megaterium*	EU723827	535/535 (100%)
84	1	*Bacillus pumilus*	EU660365	530/533 (99%)
91	1	*Bacillus pumilus*	EU231627	528/533 (99%)
Gammaproteobacteria				
22	1	*Enterobacter* sp.	DQ821735	409/435 (94%)
51	1	*Enterobacter* sp.	AF348161	520/527 (98%)
40	2	*Erwinia* sp.	EF522135	512/527 (97%)
23	2	*Erwinia* sp.	EF522135	506/525 (96%)
45	2	*Escherichia* sp.	DQ013851	523/527 (99%)
12	2	*Klebsiella oxytoca*	AB353048	514/524 (98%)
57	5	*Pantoea agglomerans*	EU598802	522/527 (99%)
5	1	*Pantoea ananatis*	AF364847	522/526 (99%)
35	4	*Pantoea* sp.	AY336554	524/527 (99%)
13	1	*Pantoea* sp.	AM909657	492/533 (92%)
14	2	*Pantoea* sp.	AY752929	506/531 (95%)
71	8	*Pantoea* sp.	AM909657	524/527 (99%)
95	2	*Pantoea* sp.	AM419023	492/531 (92%)
58	4	*Pseudomonas graminis*	PGY11150	521/521 (100%)
19	1	*Pseudomonas* sp.	EU184081	511/527 (96%)
43	6	*Pseudomonas* sp.	EU304279	512/514 (99%)
33	7	*Pseudomonas* sp.	AY464463	521/521 (100%)
15	1	*Pseudomonas* sp.	AF408925	516/521 (99%)
24	4	*Pseudomonas* sp.	EU595584	521/521 (100%)
37	1	*Pseudomonas* sp.	FM164626	489/517 (94%)
31	3	*Pseudomonas* sp.	AY131224	498/527 (94%)
46	1	*Pseudomonas* sp.	AY653220	497/525 (94%)
52	1	*Pseudomonas* sp.	EU304279	516/519 (99%)
56	1	*Pseudomonas* sp.	AM076674	519/520 (99%)
90	5	*Pseudomonas* sp.	AY014803	516/523 (98%)
54	2	*Pseudomonas* sp.	EU595584	500/527 (94%)
70	1	*Pseudomonas* sp.	AY275487	516/521 (99%)
81	1	*Pseudomonas* sp.	EU595584	520/521 (99%)
86	2	*Pseudomonas* sp.	AM111063	480/509 (94%)
87	1	*Pseudomonas* sp.	AM076674	518/520 (99%)
36	2	*Pseudomonas* sp.	DQ785814	517/521 (99%)
89	1	*Pseudomonas stutzeri*	EU603456	494/525 (94%)
