# Supplementary material for: Antimicrobial drimane sesquiterpenes and their effect on endophyte communities in the medical tree Warburgia ugandensis
Source: Front Microbiol. 2014 Feb 7;5:13. doi: 10.3389/fmicb.2014.00013 (PMC3916764; doi:10.3389/fmicb.2014.00013)
Supplement: Supplementary Data S1 — Tentative Structures of all Drimane Sesquiterpene Analytes from Warburgia ugandensis. This file provides information on which the tentative structure identification of drimane sesquiterpenes analytes is based in this study. Each analyte is presented on three pages: Page 1 contains the tentative structure with the analysis retention time, page 2 presents the MS spectrum together with the structure of the derivatized analyte, and page 3 illustrates structure fragments corresponding to specific fragments in the EI–MS spectrum. The tentative structure assignment is based on these data for each analyte respectively. The numbering corresponds to that presented in Figure 2. [file Presentation1.ZIP › Table S3.pdf]

**Table S3. Endophytic fungi from from leaves, fruits and roots of *Warburgia ugandensis*.** Sequence analysis of a partial ITS (approximately 500 bp) clone library made of isolated DNA and TRF assignment.

| Clone ID | Number of clones | T-RF [bp] | Closest identified relative (blastn) ID | Genebank ID | Similarity |
|---|---|---|---|---|---|
| **Ascomycota** | | | | | |
| Pezizomycotina; Dothideomycetes | | | | | |
| 22 | 6 | uncut | *Cladosporium* sp. | AJ279487 | 587/587 (100%) |
| 25 | 5 | uncut | *Epicoccum* sp. | AJ279463 | 579/582 (99%) |
| 66 | 1 | 88 | *Cryptococcus* sp. | AY749434 | 567/592 (95%) |
| 95 | 2 | 82 | *Sporormiella isomera* | AY943053 | 513/516 (99%) |
| Pezizomycotina; Dothioraceae | | | | | |
| 26 | 5 | 474 | *Kabatiella microsticta* | EU167608 | 611/616 (99%) |
| 60 | 4 | 93 | *Kabatiella microsticta* | EU167608 | 610/616 (99%) |
| Pezizomycotina;Eurotiomycetes | | | | | |
| 14 | 1 | 79 | *Penicillium brevicompactum* | EU833211 | 603/607 (99%) |
| 59 | 1 | 79 | *Penicillium gladioli* | DQ339568 | 616/622 (99%) |
| Pezizomycotina; Leotiomycetes | | | | | |
| 2 | 9 | 182 | *Gloeotinia temulenta* | DQ235697 | 611/611 (100%) |
| 83 | 1 | 182 | *Gloeotinia temulenta* | DQ235697 | 609/611 (99%) |
| Pezizomycotina; Pezizomycetes | | | | | |
| 74 | 2 | 151 | *Pseudaleuria quinaultiana* | EU669387 | 537/643 (83%) |
| Pezizomycotina; Sordariomycetes | | | | | |
| 1 | 8 | 426 | *Lecythophora* sp. | AY219880 | 583/608 (95%) |
| 4 | 1 | 153 | *Nigrospora oryzae* | EU272503 | 567/579 (97%) |
| 28 | 1 | 130 | *Cordyceps sinensis* | EF488439 | 151/155 (97%) |
| 46 | 2 | 135 | *Zopfiella latipes* | AY999129 | 506/585 (86%) |
| 7 | 3 | 429 | *Coniochaeta ligniaria* | AY198390. | 594/602 (98%) |
| 41 | 1 | 132 | *Fimetariella rabenhorstii* | EU781677 | 522/527 (99%) |
| 72 | 1 | 134 | *Fusarium* sp. | AF178399 | 539/544 (99%) |
| 39 | 4 | 142 | *Neurospora crassa* | FJ360521 | 615/626 (98%) |
| Saccharomycotina; Saccharomycetes | | | | | |
| 10 | 3 | uncut | *Saccharomycetales* sp. | EF060722 | 622/655 (94%) |
| 30 | 3 | 402 | *Saccharomycetales* sp. | EF060676 | 474/481 (98%) |
| 73 | 2 | uncut | *Saccharomycetales* sp. | EF060722 | 629/651 (96%) |
| 86 | 2 | 177 | *Debaryomyces hansenii* | EF196809 | 663/664 (99%) |
| **Basidiomycota** | | | | | |
| Pucciniomycotina; Microbotryomycetes | | | | | |
| 3 | 15 | uncut | *Sporidiobolus ruineniae* | EU547494 | 613/647 (94%) |
